# Freeze-thaw strength regulates carbon density through microbial assembly processes and network clustering

**DOI:** 10.1128/msystems.00067-26

**Published:** 2026-05-22

**Authors:** Shengyun Chen, Enyan Liu, Yuzheng Gu, Ali Bahadur, Lucie A. Malard

**Affiliations:** 1 Cryosphere and Eco-Environment Research Station of Shule River Headwaters, State Key Laboratory of Cryospheric Science and Frozen Soil Engineering, Northwest Institute of Eco-Environment and Resources, Chinese Academy of Sciences 824290 , Lanzhou, China; 2 College of Ecology, Lanzhou University 12426, Lanzhou, China; 3 Department F.-A. Forel for Environmental and Aquatic Sciences, University of Geneva 418814, Geneva, Switzerland; University of Guelph, Guelph, Canada

**Keywords:** microbial beta diversity, community assembly, network clusters, water-soluble organic carbon density, freeze-thaw strength

## Abstract

Freeze-thaw processes profoundly affect soil carbon storage by altering microbial communities. However, the response mechanisms of microbial community properties and their carbon sequestration potential to freeze-thaw patterns remain poorly understood, especially as climate warming intensifies freeze-thaw patterns in permafrost regions. In order to understand how freeze-thaw processes regulate microbial communities and carbon storage, we conducted *in situ* investigations during four periods of seasonal freeze-thaw processes, including thawing, completely thawed, freezing, and completely frozen periods in the surface of the active layer of alpine permafrost in the Qinghai-Tibet Plateau. We analyzed microbial community composition, beta diversity, assembly processes, co-occurrence networks, and their associations with water-soluble organic carbon density (WSOCD). Our results revealed that species turnover dominated microbial beta diversity across seasonal freeze-thaw processes. Stochastic processes dominated microbial assembly, with bacterial communities showing higher drift and fungal communities exhibiting greater dispersal limitation. Phylogenetic groups associated with assembly processes further structured microbial network clusters, and Cluster 1 was negatively correlated with WSOCD. Freeze-thaw strength regulated WSOCD by altering soil and microbial community properties, collectively explaining 37% of the observed variation. These findings provide new insights into microbially mediated carbon dynamics in alpine permafrost ecosystems, offering valuable perspectives for predicting carbon sequestration ability responses under intensified freeze-thaw patterns driven by future warming.

**IMPORTANCE:**

Understanding how seasonal freeze-thaw processes regulate microbial communities and carbon storage is vital for elucidating their interlinked dynamics in permafrost ecosystems. While microbial mediation of carbon cycling is well-established, the specific mechanisms governing microbial responses to freeze-thaw processes and their consequences for carbon sequestration remain largely unknown to date. Here, we highlight the importance of freeze-thaw processes for microbial community properties and carbon storage. These findings show important associations between microbial communities and active carbon storage, offering valuable insights for predicting how altered freeze-thaw patterns under climate warming might impact permafrost carbon storage.

## INTRODUCTION

Permafrost refers to ground materials (e.g., soil or rock) that maintain a temperature at or below freezing for a minimum of two continuous years (1). Overlying this permanently frozen ground is the active layer, which is subject to seasonal thawing and freezing, directly influencing soil processes, hydrology, and microbial activity (2). The Qinghai-Tibet Plateau (QTP) hosts the largest extent of high-altitude permafrost on Earth, representing nearly 75% of the mountain permafrost found in the Northern Hemisphere (3). Permafrost-affected soils globally store approximately 1,700 Pg of organic carbon (C), which constitutes about half of the global subsurface C stocks (4). In the QTP, the active layer contains about 14 Pg of organic C down to 3 m depth (4). This buried C was preserved due to the limited activity of microbial decomposition under persistently low temperatures and water-saturated conditions (5). However, accelerated climate warming in recent decades has driven extensive permafrost thaw (6), releasing stored C and reactivating microbial activity, which promote organic matter degradation and amplifies greenhouse gas emissions (7). Within this context, water-soluble organic carbon (WSOC) represents a highly reactive and mobile form of soil C that fuels microbial activity (8). It serves as an immediately available substrate for microorganisms, rapidly moving through the soil profile to support respiration and enzyme production (8). The production of WSOC is considered to be facilitated by microbial activity (9), and the qualitative and quantitative aspects of this C fraction are crucial for understanding C cycling processes (10). Therefore, these tightly coupled WSOC and microbial interactions play a central role in regulating carbon cycling in permafrost ecosystems.

In the QTP permafrost regions, the surface of the active layer undergoes repeated freeze-thaw cycles driven by diurnal or seasonal thermal dynamics (11). Meta-analyses and laboratory simulation research have confirmed that freeze-thaw events substantially affect soil microstructure, particularly aggregate stability and porosity (12). Together, these freeze-thaw-induced stresses act as a strong environmental filter that affects microbial survival strategies (13, 14). For instance, physical stresses (e.g., ice crystal formation and osmotic fluctuation) are known to selectively reduce microbial biomass and impair microbial carbon and nitrogen metabolic functions (15), thereby considerably influencing microbial community properties (15–17). Additionally, these physical processes powerfully regulate WSOC. Indeed, soil aggregate disruption and microbial cell lysis can release labile WSOC during thaw, whereas sustained microbial respiration can deplete this labile pool, leading to either transient increases or decreases (18). With ongoing climate warming, freeze-thaw patterns are anticipated to experience more pronounced variations, with cascading effects on terrestrial ecosystems (11). Observations over recent decades have shown substantial alteration in freeze-thaw patterns, including delayed onset of the freezing period (FP), earlier ending of the FP, and earlier onset of the thawing period (TP) (19). Consequently, the completely frozen period (CFP) duration is shorter, while the completely thawed period (CTP) is longer. Furthermore, experimental warming studies and model simulations project that freeze-thaw strength (FTS), which reflects the frequency and magnitude of soil temperature fluctuations around the freezing point (e.g., 0°C), is expected to rise in the future (20). Although recent *in situ* studies on the QTP have linked higher FTS to greater microbial richness and soil multifunctionality (21), most existing work considered microbial communities or WSOC dynamic separately and often relied on simulations. Consequently, how seasonal freeze-thaw processes jointly regulate microbial community dynamics and WSOC remains poorly understood.

A core aspect of microbial ecology is understanding the processes shaping microbial community assembly (22, 23). Current ecological theories propose that community assembly is governed by two fundamental processes (24). Deterministic processes, aligned with niche-based theory, describe cases where microbial distribution is shaped by environmental filtering, such as pH, temperature, and nutrient gradients, along with species interactions, including competition, facilitation, mutualism, and predation (22). Conversely, stochastic processes, derived from neutral theory, attribute microbial distribution to random demographic events including birth, migration, and death (24). While deterministic processes are key in permafrost-affected soils (25, 26), in thawing permafrost, stochastic processes dominate short-term microbial assembly before shifting back to deterministic control over longer timescales (17). Importantly, the nature of these assembly processes not only affects composition stability (27) but also directly influence the ecosystem C balance by selecting for microbial taxa with distinct functional attributes (17, 28). Earlier research demonstrated that microbial community organization is influenced by assembly processes, with specific taxa predominantly contributing to chemoheterotrophic metabolism functions (29). Integrating community assembly processes with species co-occurrence patterns provides valuable insight into the fundamental mechanisms that govern microbial community structure (24, 30). For instance, a study on the QTP revealed that network connectivity and modularization exhibit distinct response patterns under different moisture conditions (31). However, the interplay between assembly processes, network topology, and C dynamics during seasonal freeze-thaw cycles remains poorly understood. Elucidating these interactions is crucial for predicting permafrost C cycle responses to climate change.

In this study, we build upon recent work showing that stronger FTS enhanced microbial stability and strengthened the diversity-soil multifunctionality relationship (21). Using the same data set of soil samples collected from the surface of the active layer in the QTP alpine permafrost region, including topsoil (0–10 cm) and subsoil (10–20 cm), during distinct seasonal freeze-thaw periods (TP, CTP, FP, and CFP), we examined how freeze-thaw processes regulate microbial communities and carbon storage. Our analyses are designed to answer three main questions. (i) How do seasonal freeze-thaw processes affect the compositions and beta diversities of bacterial and fungal communities? (ii) What are the dominant assembly processes (deterministic or stochastic processes) of microbial communities in different freeze-thaw periods and at two depths? (iii) What are the underlying mechanisms of microbial-mediated seasonal variations in water-soluble organic carbon density (WSOCD)? To answer these questions, we integrated beta diversity decomposition, phylogenetic bin-based null model analysis (iCAMP), and phylogenetic bin-based network analysis to assess how seasonal freeze-thaw processes shape microbial community composition, beta diversity, assembly processes, co-occurrence network patterns, and their relationships with WSOCD.

## RESULTS

### Composition and beta diversity of microbial communities

**Fig 1:**
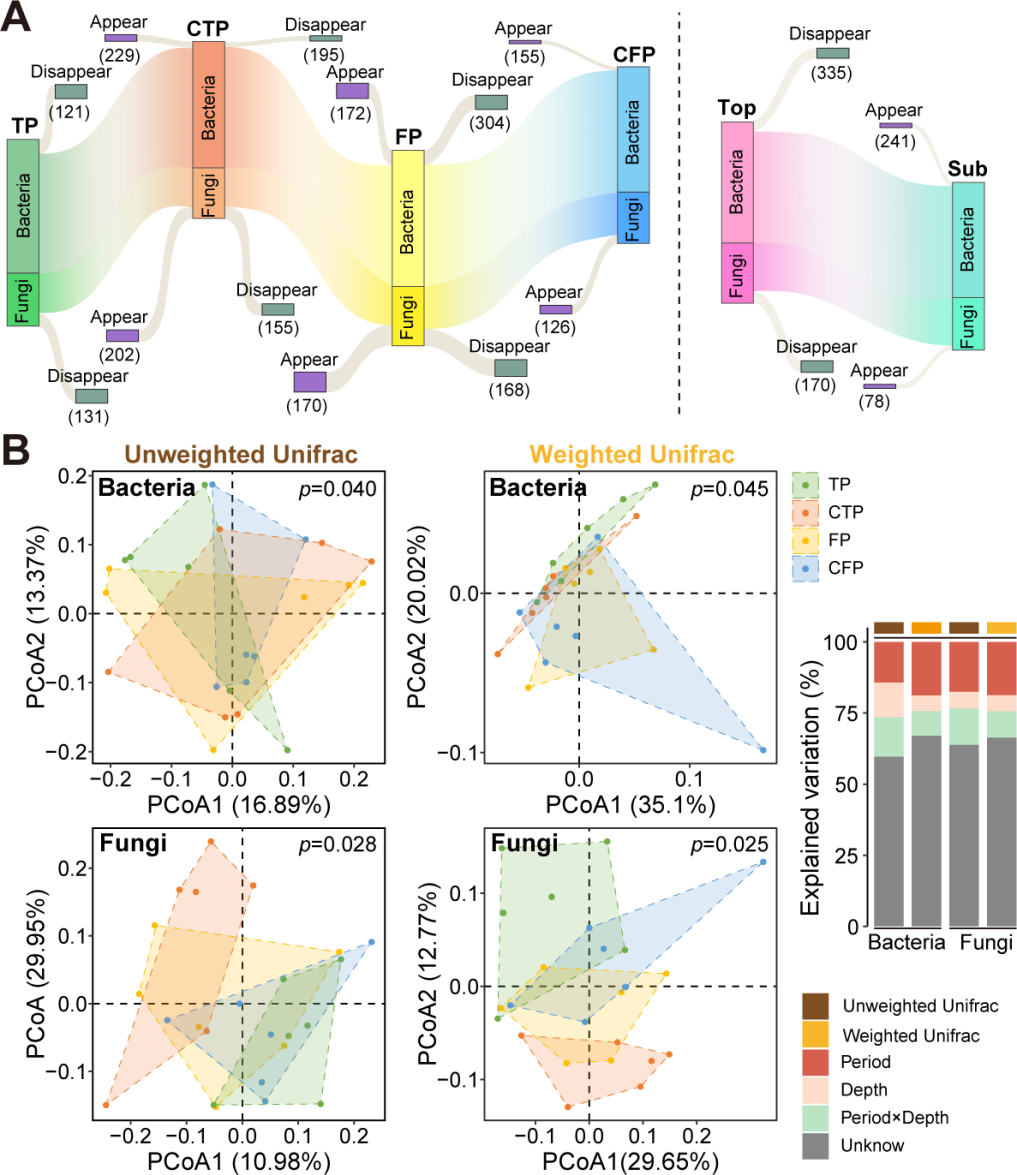
Composition of microbial communities during four periods of seasonal freeze-thaw processes and at two depths. (**A**) Changes in bacterial and fungal operational taxonomic units, depending on periods of seasonal freeze-thaw processes and depths. (**B**) Principal coordinate analysis (PCoA) and permutational multivariate analysis of variance on bacterial and fungal communities during seasonal freeze-thaw processes based on unweighted and weighted UniFrac distances. Bar charts show the explained variations by period, depth, and their interactions. TP, CTP, FP, and CFP represent the thawing, completely thawed, freezing, and completely frozen periods, respectively. Top and Sub represent topsoil (0–10 cm) and subsoil (10–20 cm), respectively.

Microbial operational taxonomic units (OTUs) showed dynamic turnover across the four seasonal freeze-thaw periods and at both depths. Notably, more bacterial OTUs disappeared from TP to CTP and from FP to CFP, while more OTUs appeared from TP to CFP; a comparable pattern was also evident in the fungal communities, indicating stronger temporal species replacement rather than simple species loss. The species turnover of microbial communities was more pronounced from CTP to FP and from FP to CFP (Fig. 1A). We also observed that a greater number of OTUs disappeared from the topsoil to the subsoil, highlighting a decrease in taxa with depth (Fig. 1A). Consistent with these patterns, microbial community composition also shifted significantly, as indicated by principal coordinate analysis (PCoA), in conjunction with global and pairwise permutational multivariate analysis of variance using both weighted and unweighted UniFrac distances (Fig. 1B; Table S1). However, bacterial community differed significantly between topsoil and subsoil, with the thawing/freezing period demonstrating the strongest effect (Fig. 1B; Fig. S1; Table S1). Nonetheless, the combined effects of period and depth explained less than 50% of the observed variation, indicating that additional factors contributed to community dynamics. In addition, unweighted UniFrac distances explained a greater proportion of the variation than weighted distances (Fig. 1B), suggesting that changes were predominantly driven by alterations in species composition rather than abundance.

**Fig 2:**
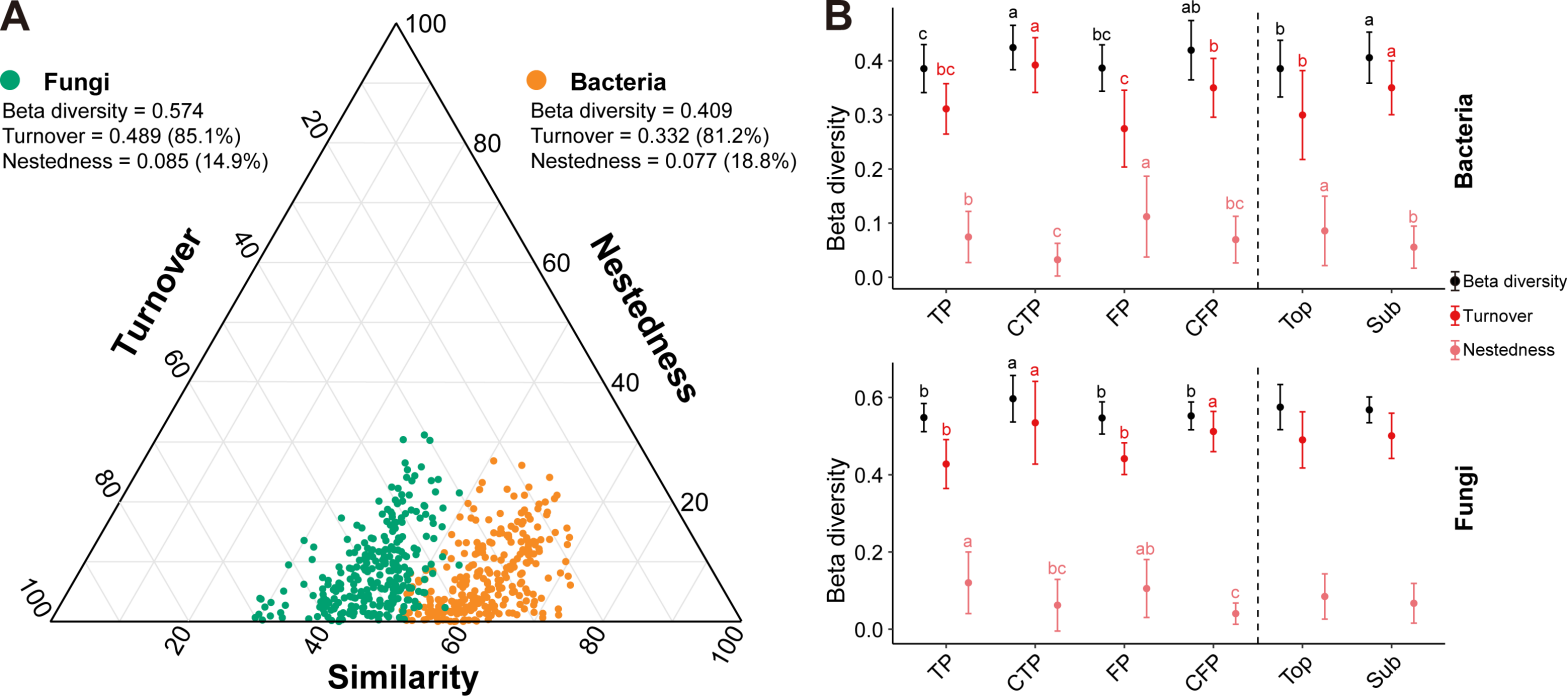
Beta diversity of microbial communities during four periods of seasonal freeze-thaw processes and at two depths. (**A**) Ternary diagram of bacterial and fungal beta diversity components, including species turnover and nestedness. Beta diversity = species turnover + nestedness, and similarity = 1 – beta diversity. (**B**) Variations of beta diversity components in four periods and at two depths. Lowercase letters represent significant differences among the four periods or between the two depths (*P* < 0.05). TP, CTP, FP, and CFP represent the thawing, completely thawed, freezing, and completely frozen periods, respectively. Top and Sub represent topsoil (0–10 cm) and subsoil (10–20 cm), respectively.

To analyze patterns in microbial beta diversity, we decomposed it into species turnover and nestedness constituents. Species turnover accounted for the majority of beta diversity, contributing 81.2% in bacterial communities and 85.1% in fungal communities, while nestedness contributed only 18.8% and 14.9%, respectively (Fig. 2A). Both microbial communities exhibited the highest beta diversity and turnover in CTP, with the lowest value in FP, whereas nestedness showed an opposite pattern. Regarding the soil depth, bacterial beta diversity and turnover were significantly higher in subsoil, whereas nestedness was more pronounced in topsoil. Fungal communities showed no significant depth-dependent variations in beta diversity (Fig. 2B).

### Microbial assembly processes

**Fig 3:**
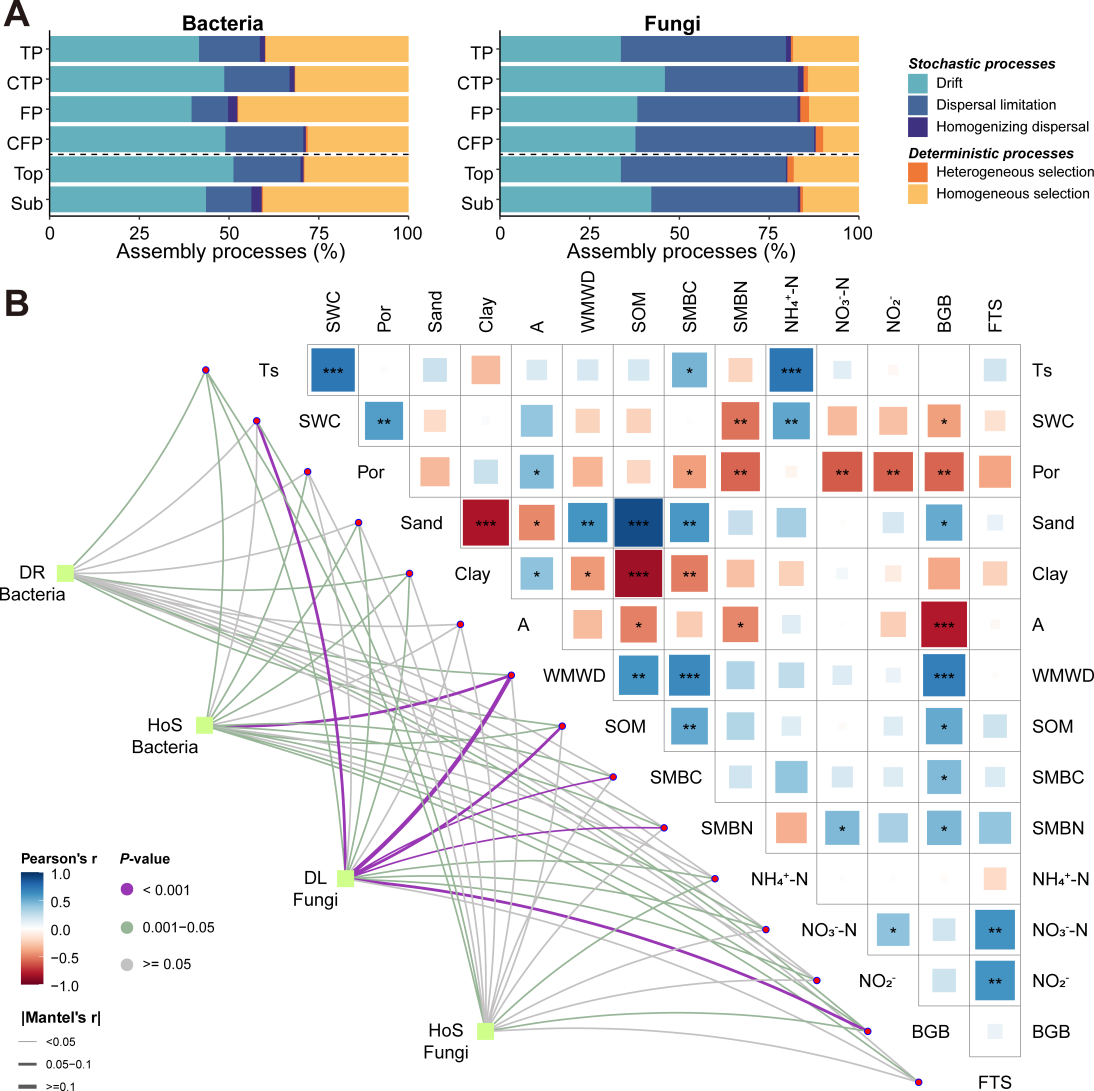
Relative importance of assembly processes during four periods of seasonal freeze-thaw processes and at two depths, and correlations between environmental variables and assembly processes in microbial communities. (**A**) Relative importance of assembly processes in the bacterial and fungal communities during four periods of seasonal freeze-thaw processes and at two depths. (**B**) The modified Mantel test for stochastic and deterministic processes in microbial communities. Asterisks indicate statistical significance (*** *P* < 0.001, **P < 0.01, and *P < 0.05). Ts, soil temperature; SWC, soil water content; Por, soil porosity; A, aggregate content; WMWD, wet-sieving mean weight diameter; SOM, soil organic matter; SMBC, soil microbial biomass carbon; SMBN, soil microbial biomass nitrogen; BGB, belowground biomass; FTS, freeze-thaw strength. TP, CTP, FP, and CFP represent the thawing, completely thawed, freezing, and completely frozen periods, respectively. Top and Sub represent topsoil (0–10 cm) and subsoil (10–20 cm), respectively.

Stochastic processes were the principal mechanism that dominated the structuring of both bacterial and fungal communities throughout the four seasonal freeze-thaw periods and at both depths investigated, yet bacterial and fungal communities responded through different mechanisms (Fig. 3A). Homogeneous selection served as the primary driver of deterministic assembly within bacterial communities, whereas drift exerted a dominant influence on stochastic assembly. The impact of drift was higher in CTP and CFP, whereas homogeneous selection showed greater importance in TP and FP (Fig. 3A). In fungal communities, both drift and dispersal limitation were key stochastic processes, with homogeneous selection dominating the deterministic processes. Notably, drift was most pronounced in CTP, while homogeneous selection was lowest in CFP. In terms of soil depth, bacterial communities exhibited higher drift and lower homogeneous selection in topsoil, but fungal communities showed the opposite pattern (Fig. 3A). Consistent with these patterns, bacterial communities demonstrated greater migration rates, indicating that fungal communities exhibited a stronger influence of dispersal limitation compared to their bacterial counterparts (Fig. S2). Moreover, bacterial community similarity decreased more sharply with increased environmental distance (environmental dissimilarity calculated as Euclidean distance of standardized soil properties) than the fungal community, suggesting that fungal communities were more resistant to environmental filtering (Fig. S3).

A total of 3,453 bacterial OTUs were divided into 185 phylogenetic bins, and 1,778 fungal OTUs were classified into 98 bins (Fig. S4 and S5). For bacterial communities, drift dominated 65% (47% relative abundance), and homogeneous selection governed 23% (47% relative abundance). Fungal communities showed distinct patterns, with drift (67% bins, 39% relative abundance) and dispersal limitation (23% bins, 36% relative abundance) prevailing, while homogeneous selection dominated only 8% of the bins (24% relative abundance). Proteobacteria, Bacteroidetes, and Acidobacteria bins were primary bacterial contributors to drift, with Bacteroidetes bins also significantly associated with homogeneous selection (Fig. S4 and S6A). In fungal communities, Ascomycota and Zygomycota bins predominantly exhibited drift, while Ascomycota bins additionally contributed to homogeneous selection (Fig. S5 and S6B). Assembly processes were significantly associated with most measured environmental factors (Fig. 3B). It is worth noting that the dispersal limitation process of fungal communities demonstrated marked correlations with multiple factors, including soil water content (SWC), aggregate stability, and belowground biomass (BGB), whereas in bacterial communities, aggregate stability (wet-sieving mean weight diameter [WMWD]) showed the strongest correlation with homogeneous selection process. Interestingly, FTS was only weakly correlated with assembly processes (Fig. 3B).

### Network analysis of microbial co-occurrence patterns

**Fig 4:**
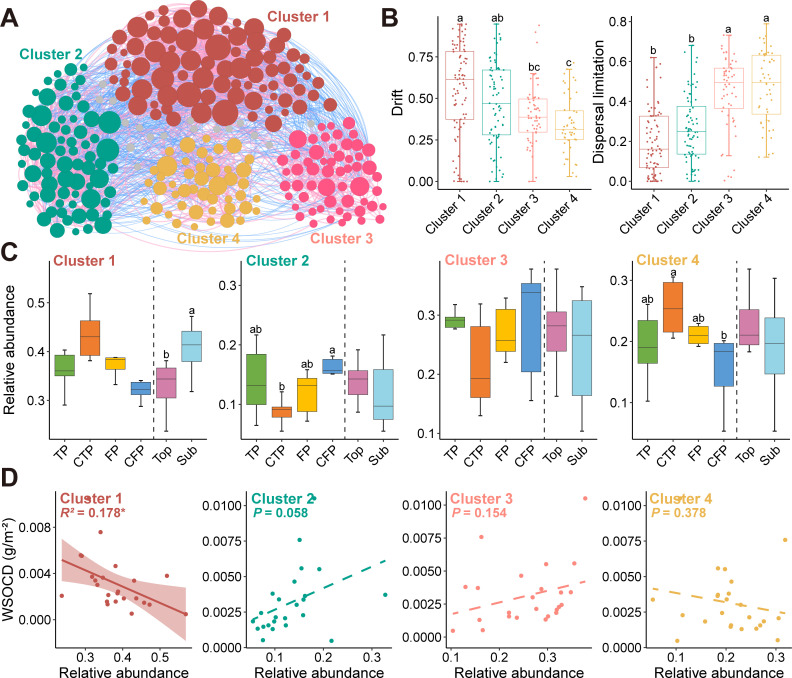
Microbial co-occurrence network and its association with water-soluble organic carbon density (WSOCD). (**A**) Co-occurrence network of microbial communities. Node colors denote distinct network clusters, while node size reflects the relative abundance of bins. Edges represent significant co-occurrence relationships (|*R*| > 0.6, *P* < 0.05); edge color indicates positive (red) and negative (blue) correlations. The resulting network contained 283 nodes and 1,665 edges (comprising 1,111 positive and 554 negative edges) and showed high modularity (1.233). (**B**) Differences in the assembly processes of distinct clusters. (**C**) Comparison of the relative abundance of different clusters during four periods of seasonal freeze-thaw processes and at two depths. (**D**) The relationships between the relative abundance of different clusters and WSOCD. Lowercase letters represent significant differences among the four periods or between the two depths (*P* < 0.05). Asterisks indicate statistical significance (*** *P* < 0.001, **P < 0.01, and *P < 0.05). TP, CTP, FP, and CFP represent the thawing, completely thawed, freezing, and completely frozen periods, respectively. Top and Sub represent topsoil (0–10 cm) and subsoil (10–20 cm), respectively.

The phylogenetic bins-based co-occurrence interkingdom network revealed distinct bacterial- and fungal-dominated clusters that differed in their assembly processes (Fig. 4A). The network comprised 283 nodes and 1,665 edges, with bacterial bins representing 65.9% of the total network. The co-occurrence network exhibited a high degree of modularity, separating into four distinct ecological clusters (Clusters 1–4) that corresponded to specific assembly processes (Fig. 4A). Clusters 1 and 2 were predominantly bacterial bins (92.5% and 90.2%, respectively) and were mainly affiliated with the phyla Proteobacteria, Planctomycetes, and Bacteroidetes. In contrast, Clusters 3 and 4 were dominated by fungal bins (80.4% and 60.5%, respectively) and were primarily composed of taxa belonging to Ascomycota and Basidiomycota (Tables S2 and S3). Clusters 1 and 2 exhibited higher drift levels and lower dispersal limitation compared to Clusters 3 and 4. Selection patterns also varied distinctly among clusters, with Cluster 4 showing greater heterogeneous selection than Cluster 1 and Cluster 2 exhibiting stronger homogeneous selection than Cluster 3 (Fig. 4B; Fig. S7). This is in line with our previous observations that bacteria are more impacted by drift, while fungi are more impacted by dispersal limitation (Fig. 3A). Additionally, the clusters displayed unique patterns in response to seasonal and vertical changes. Cluster 2 exhibited a greater relative abundance during the CFP than the CTP, whereas Cluster 4 exhibited the opposite trend. Additionally, Cluster 1 showed increased relative abundance in subsoil compared to the topsoil (Fig. 4C).

### Relationships among microbial communities, environmental variables, and WSOC storage

**Fig 5:**
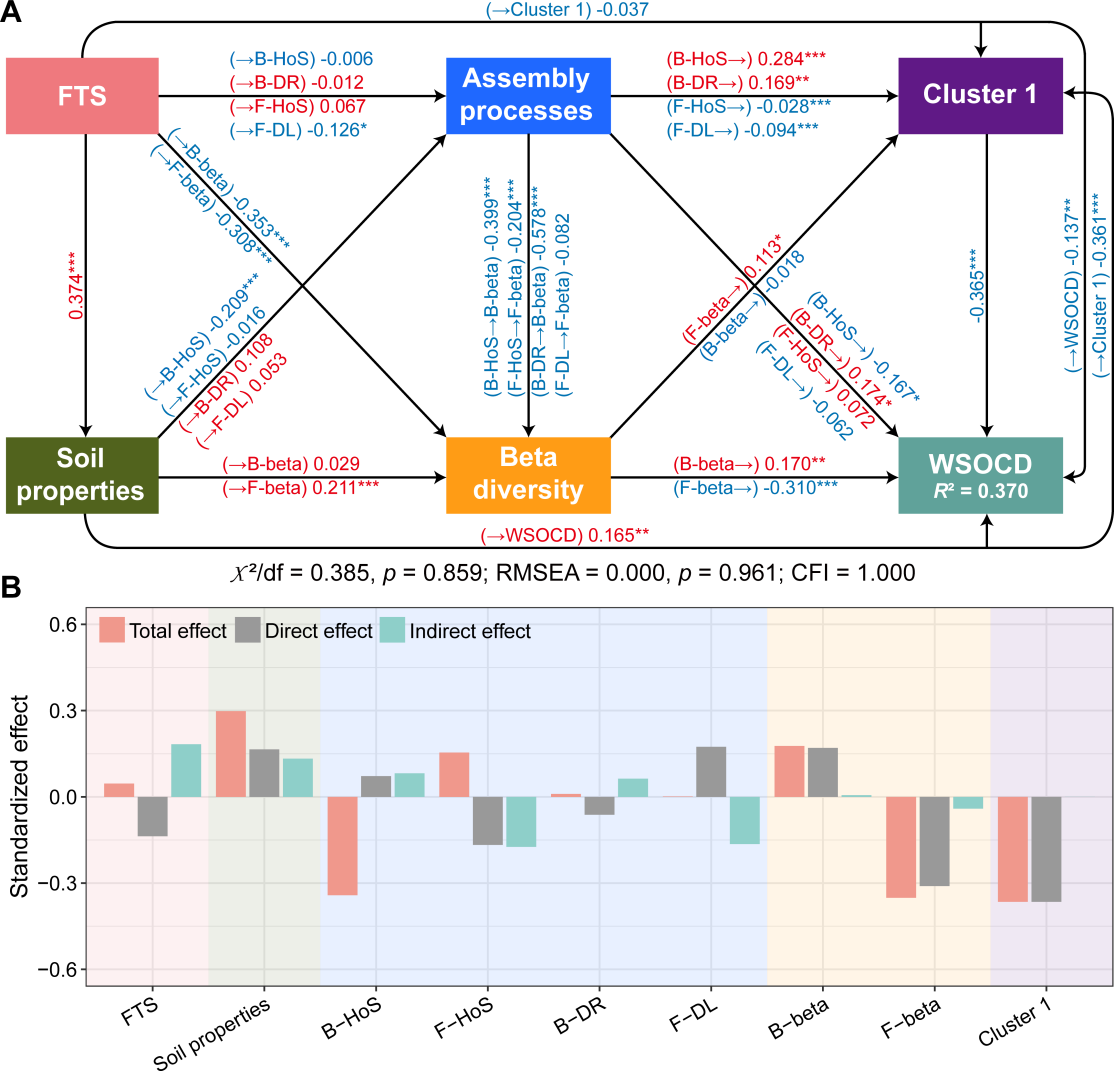
Effects of environmental variables and microbial communities on water-soluble organic carbon density (WSOCD). (**A**) The structural equation model shows the direct relationships among environmental variables, microbial assembly processes, beta diversity, network cluster, and WSOCD. To simplify the graphs, variables with the same category are grouped into the same boxes in the model; however, these boxes do not represent latent variables. Numbers adjacent to arrows indicate the effect size of the relationship. *R*^2^ denotes the proportion of variance explained. Asterisks indicate statistical significance (***P < 0.001, **P < 0.01, and *P < 0.05). (**B**) Bar graph shows the standardized effect on WSOC, encompassing total, direct, and indirect effects. B-beta, beta diversity of bacterial communities; B-DR, drift of the bacterial community; B-HoS, homogeneous selection of bacterial communities; F-beta, beta diversity of fungal communities; F-DL, dispersal limitation of the fungal community; F-HoS, homogeneous selection of fungal communities; FTS, freeze-thaw strength.

WSOCD was significantly higher in CFP and topsoil (Fig. S8). Regression analysis indicated a negative correlation between WSOCD values and the relative abundance of Cluster 1 (Fig. 4D). The structural equation model (SEM) indicated that FTS influenced soil properties, which subsequently affected microbial assembly processes and beta diversity, ultimately shaping Cluster 1 (Fig. 5A). The model explained 37% of the WSOCD variation, soil properties demonstrated the largest positive influence, while FTS exhibited a direct negative influence on WSOCD. In terms of assembly processes, bacterial homogeneous selection exerted stronger influence on WSOCD than the fungal community. Additionally, bacterial beta diversity correlated positively with WSOCD, in contrast to a negative association observed for fungal beta diversity. These results highlight distinct regulatory roles of bacterial and fungal communities in WSOCD dynamics. Notably, FTS exerted a positive total effect on WSOCD through its effects on soil properties and microbial community properties (Fig. 5B).

## DISCUSSION

This study investigated the impact of seasonal freeze-thaw processes on microbial community composition and assembly, as well as their influence on carbon dynamics. Consistent with previous work in permafrost regions (15, 21), microbial communities in the QTP were highly sensitive to freeze-thaw processes. We showed pronounced shifts in microbial community composition during the four seasonal freeze-thaw periods and at both depths. Consistent with previous work in permafrost regions, microbial communities on the QTP were highly sensitive to freeze-thaw processes (15, 21). Under freezing conditions in alpine environments, reduced soil water availability and elevated osmotic pressure can physically damage microbial cells, selectively removing less tolerant microorganisms (32). This selective pressure favors cold-tolerant microorganisms and restructures community composition (33). Consistent with this conceptual framework, our results showed that Cluster 2, which exhibited a greater relative abundance in the CFP, was predominantly composed of Proteobacteria, Planctomycetes, and Bacteroidetes. These phyla are widely recognized to harbor psychrotolerant taxa capable of surviving low temperatures and water stress in permafrost environments through metabolic plasticity and dormancy strategies (5, 33). This suggests that specific groups of microbes co-respond to freeze-thaw stress through shared survival strategies, functioning as a coherent unit to regulate carbon dynamics even under extreme conditions. We also observed the influence of freeze-thaw cycles across soil layers, leading to significant shifts in microbial community composition and beta diversity with depth. Previous research in northern China has reported the highest frequency of such cycles at a depth of 0–5 cm (34), indicating that topsoil microbial communities are subject to greater environmental disturbance from freeze-thaw events, resulting in differences in community composition along the vertical profile.

Overall, our findings demonstrated that species turnover, rather than nestedness, served as the dominant component of beta diversity in both bacterial (81.2%) and fungal (85.1%) communities, indicating substantial compositional shifts mediated by environmental filtering (35). This species turnover pattern likely reflects increased environmental heterogeneity due to hydrothermal fluctuations during freeze-thaw processes (36) and ecological niches redistribution (37) that collectively drive species replacement. Importantly, these dynamics offer a mechanistic explanation for our previous findings, where stronger freeze-thaw strength was linked to higher microbial richness (21). The substantial appearance of taxa observed here (Fig. 1A) suggests that freeze-thaw disturbance does not merely act as a destructive filter; rather, it creates transient niches that facilitate the colonization of diverse opportunistic or stress-tolerant species, thereby sustaining high microbial richness despite the environmental stress. Indeed, freeze-thaw cycles disrupt soil aggregates, releasing nutrients and creating new ecological niches that selectively enhance microbial activity and favor certain taxa. At the same time, microorganisms adapted to prolonged subzero conditions may be disadvantaged or eliminated when exposed to warmer temperatures (38). Interestingly, we observed a reduced turnover during stable conditions in the CTP and CFP, consistent with the expectation that turnover decreases when environmental heterogeneity decreases (39), further confirming that freeze-thaw induced environmental fluctuations are the primary driver of community renewal.

The analysis of assembly process indicated that these turnover patterns emerge from a combination of stochastic and deterministic mechanisms. Stochastic processes dominated overall microbial community assembly. Bacterial communities exhibited higher drift levels, whereas fungal communities showed greater dispersal limitation and high ecological drift. Ecological drift can strongly influence community composition, leading to high temporal variability (40). This dominance of stochasticity implies that microbial response to freeze-thaw events is driven less by competitive exclusion (deterministic) and more by the random survival of dormant cells and physical isolation. In contrast, the pronounced dispersal limitation in fungi was closely associated with BGB, suggesting that greater root biomass creates physical barriers in soil that restrict fungal movement and intensify dispersal limitation (41, 42). Such restriction enhances species turnover by limiting the exchange of taxa among habitats, causing communities to diverge even in similar environmental conditions (43). These contrasting patterns likely reflect fundamental biological differences between bacteria and fungi. Bacteria, with their short life cycles, rapid reproduction, and broad physiological flexibility, are more strongly influenced by stochastic processes such as drift, which may facilitate rapid colonization from regional species pools (44). Fungi, by contrast, often rely on longer-distance dispersal via spores, a process that may be particularly constrained during the CFP, further reinforcing dispersal limitation (45). These results are in line with recent studies showing that stochasticity is particularly important in thawing permafrost, where frequent disturbance and spatial isolation weaken the strength of environmental filtering (17).

Previous work has shown that bacterial-fungal interactions, including antibiotic-mediated antagonism and symbiotic decomposition of complex organic substrates, can profoundly influence community structure and co-occurrence networks (46, 47). In frozen environments, seasonal freeze-thaw cycles can weaken environmental filtering by creating highly dynamic and heterogeneous soil conditions, consequently lowering the influence of deterministic processes. Under spatially heterogeneous or physically isolated conditions, stochastic processes can outweigh deterministic forces (22, 24), consistent with our observation that stochastic processes played a dominant role in community assembly. Despite this overall pattern, different phylogenetic groups followed distinct assembly processes, resulting in ecologically separated clusters. Our cross-kingdom co-occurrence network further indicated that phylogenetic groups with different assembly processes tend to aggregate into distinct modules. Specifically, Clusters 1 and 2 exhibited higher drift, while Clusters 3 and 4 were more constrained by dispersal limitation. The coexistence of symbiotic interactions that promote growth and antagonistic interactions that exclude competitors likely reinforces the phylogenetic divergence and generates modular network structure (47). Moreover, differential environmental adaptation among microorganisms drives the assembly of ecologically similar taxa into separate clusters (48) and contributes to the observed beta diversity patterns. These findings highlight that assembly processes, mediated by species interactions and environmental filtering, are pivotal in structuring microbial co-occurrence networks.

WSOC primarily originates from plant litter, decaying biomass, root exudates, and microbial residues (49). Its concentration is regulated by multiple factors (e.g., precipitation, soil temperature, moisture, and microbial populations) (49). Our results indicated that FTS is negatively associated with WSOCD, and this relationship is likely mediated by two mechanisms. First, freeze-thaw processes disrupt soil aggregate structure and release nutrients (28), thereby stimulating microbial respiration and accelerating the rate of WSOC loss (50, 51). Second, previous work has shown that microbial functional gene abundance and alpha diversity, which are enhanced by permafrost thaw, can accelerate soil C release, amplifying the reduction of unstable WSOC (52). The SEM results suggest that similar mechanisms operate during the seasonal freeze-thaw processes, with stronger FTS associated with faster WSOC decomposition while also exerting indirect positive effects on WSOCD through its influence on soil properties and microbial community attributes. Notably, the negative association between Cluster 1 and WSOCD highlights the value of network approaches for linking microbial community structure to soil functions (53). Furthermore, bacterial beta diversity exhibited a significantly positive relationship with WSOCD, whereas fungal beta diversity showed a negative association. This suggests that the loss of WSOC may be substantially severe when the fungal community composition is more diverse, while WSOC sequestration is greater with larger variations in bacterial community composition. These patterns align with known functional differences. For instance, bacteria preferentially use hydrophilic C, while fungi prefer to process the hydrophobic fraction (54), and mycorrhizal reduction correlates with WSOC accumulation (55). These findings suggest different ecological roles for bacteria and fungi in regulating WSOC. Since WSOCD is regarded as a vital indicator of soil C pool quality and stability (56), fluctuations in WSOC dynamics are likely to exert strong influences on organic C sequestration, thereby affecting climate feedbacks.

Our results further suggest that stochastic assembly, species turnover-driven beta diversity, and network-mediated carbon regulation are key processes during seasonal freeze-thaw processes. Although our study is based on a single year of *in situ* observations, they represent the essential seasonal dynamics that will be increasingly intensified under climate warming and likely to be generalizable to longer-term climate-driven changes. Importantly, our study highlights FTS as a pivotal driver regulating ecosystem dynamics, rather than merely a seasonal descriptor. Unlike broad seasonal categories, FTS integrates both the frequency and magnitude of temperature fluctuations, providing a quantitative metric of the “environmental filtering strength” exerted on microbial communities. Meanwhile, because FTS is intrinsically tied to the presence of freeze-thaw processes and is therefore non-zero only in TP and FP, our field design cannot completely separate the effects of FTS from the broader seasonal context. Accordingly, the effects of FTS inferred from the SEM should be interpreted as process-based associations, rather than as results of a fully controlled comparison under identical background conditions. Despite this limitation, our study provides a valuable baseline for understanding and predicting how microbial assembly and C cycling may respond to more frequent and stronger freeze-thaw patterns in the future. As climate warming accelerates and freeze-thaw events increase in both frequency and magnitude, these microbial mechanisms are expected to become even more influential in shaping soil carbon stability.

### Conclusions

This study examined seasonal freeze-thaw processes in the surface of the active layer of permafrost-affected soils in the QTP. We characterized shifts in microbial community composition, beta diversity patterns, assembly processes and their linkages with WSOCD. Our results revealed that species turnover, rather than nestedness, dominated microbial beta diversity patterns. Stochastic processes (particularly drift and dispersal limitation) governed both bacterial and fungal community assembly during seasonal freeze-thaw processes. Different phylogenetic groups exhibited distinct assembly processes, further affecting the clustering of their co-occurrence patterns. Network modularity reflected fundamental ecological trade-offs: the bacteria dominated clusters thrive under drift-dominated stochasticity, whereas fungal-dominated clusters were constrained by dispersal limitation, demonstrating that assembly processes are pivotal in structuring microbial co-occurrence networks. Importantly, bacterial and fungal communities regulated WSOCD through divergent mechanisms, with FTS exerting an overall positive effect. Collectively, these findings highlight new insights into the mechanistic links between microbial community properties and WSOCD, which can aid in assessing and predicting changes in C storage and the potential ecological consequences that may arise from altered freeze-thaw patterns under future warming.

## MATERIALS AND METHODS

### Study area description

The study area is an alpine meadow ecosystem located within the permafrost zone of the Shule River headwaters observation site (98.27° E, 38.35° N, altitude: 4,014 m) in the western Qilian Mountains on the northeastern margin of the QTP. The area experiences pronounced freeze-thaw processes at the surface of the active layer. The local climate is characterized by annual precipitation of 359 mm and an annual temperature averaging −3.5°C. From 2001 to 2020, this region experienced a warming and wetting trend, with the yearly precipitation rising at a rate of 5.84 mm per year and the mean annual air temperature increasing at a rate of 0.02°C per year (57). The soils of the study area are classified as cold calcic soil (kastanozems, Chinese Soil Classification System), and the dominant vegetation consists primarily of *Kobresia pygmaea* and *K. humilis* (58). The region contains sub-stable alpine permafrost, with an active layer extending to approximately 2.3 m in thickness (58).

### Fieldwork and laboratory analyses

An *in situ* study was conducted in 2013, targeting four distinct periods of seasonal freeze-thaw processes: the thawing period (28 April and 4 May), completely thawed period (1 August), freezing period (29 September and 5 October), and completely frozen period (31 December). We collected a total of 30 soil samples from the surface of the active layer at two depths: topsoil (0–10 cm) and subsoil (10–20 cm). It is noteworthy that samples collected twice at 0–10 cm depth during the TP and FP were combined into one sample for subsequent analysis. Finally, our data set consisted of 24 composite samples. Details of the design and setup of the experimental sampling have been previously described (21).

The soil samples were separated into three subsamples for different analyses. The first portion was stored frozen at −80°C for the extraction of subsequent genomic DNA. A second portion was also frozen for the assessment of dry-sieving mean weight diameter (DMWD), WMWD, pH, redox potential (Eh), WSOC, NH_4_^+^, NO_3_^−^, soil microbial biomass nitrogen (SMBN) and soil microbial biomass carbon (SMBC), and aggregate content (A). The third portion was air-dried for the measurement of major ions (NH_4_^+^, NO_3_^−^, and NO_2_^−^), total nitrogen (TN), and total carbon (TC). Soil bulk density (BD) was determined with a 100 cm^3^ cutting ring. Concurrently, SWC and soil temperature (Ts) at both depths were recorded automatically at 10-minute intervals using a Hydra Probe II sensor (Stevens) connected to a CR1000X datalogger (Campbell), with data collection initiated in July 2011.

Soil pH and Eh levels were analyzed in a 1:5 soil-water mixture using a PHBJ-260 pH meter (INESA) equipped with composite electrodes. TN was determined by the micro-Kjeldahl procedure. Particle size distribution (sand and clay) was assessed through the wet-sieving techniques. BGB was collected using a 4.8 cm diameter corer and sieved (2 mm) to remove impurities. All biomass materials were dried at 80°C until a constant weight was achieved. SMBN and SMBC were determined employing the chloroform fumigation-extraction technique. Ion analysis was performed using ion chromatography: NH_4_^+^ ion was quantified with a DX-600 system (Dionex, USA), while SO_4_²^−^, NO_2_^−^, and NO_3_^−^ ions were analyzed using an ICS-2500 System (Dionex) following the manufacturer’s protocols. BD, soil organic matter, soil porosity, A, TC, C:N, WSOCD, and aggregate stability (DWMD and WMWD) have been documented in prior studies (20, 21, 59, 60).

### Sequencing and bioinformatics

Microbial community analysis followed an established sequencing protocol (21). In brief, soil samples were used to extract genomic DNA using the PowerSoil DNA isolation kit. The bacterial 16S rRNA genes were amplified with the 515F/907R primer set (515F: GTGCCAGCMGCCGCGGTAA, 907R: CCGTCAATTCMTTTRAGT), and fungal internal transcribed spacer (ITS) gene regions were amplified using the ITS1/ITS2 primers (ITS1F: CTTGGTCATTTAGAGGAAGTAA, ITS2: GCTGCGTTCTTCATCGATGC). Sequencing was carried out on the Illumina MiSeq platform (utilizing paired-end 250 and 300 bp modes) at BGI (Wuhan, China). A minimum of 50 Mbp of raw sequencing data were generated per sample.

Bioinformatics processing of the raw sequence data was conducted using QIIME (v1.9.1) (61), FLASH (v1.2.11) (62), and USEARCH (v7.0.1090) (63); the detailed pipeline is provided in the Supplemental Material. To maintain a balanced sample size across different freeze-thaw periods, data from replicate samples (collected twice at the 0–10 cm depth during the TP and the FP) were resampled during the bioinformatic analysis.

### Statistical analysis

In this study, we followed previous studies and used 0.0°C as a threshold for Ts to classify each day into three types: (i) completely thawed days, identified when the daily minimum Ts exceeded 0.0°C; (ii) completely frozen days, occurring when the daily maximum Ts remained at or below 0.0°C; and (iii) freeze-thaw days, defined by a daily minimum Ts of ≤0.0°C and a daily maximum Ts of >0.0°C (20, 21). The actual freezing point of soil water may deviate slightly from 0°C due to solutes and soil matrix effects, so this threshold represents a practical convention. These processes were further divided into four distinct periods: TP, CTP, FP, and CFP (21, 64). The onset of each period was defined by three consecutive days of the respective freeze-thaw state. To quantify the strength of freeze-thaw events, FTS was calculated for each freeze-thaw day. This index integrates both the frequency distribution of positive and negative Ts values and the cumulative sums of positive and negative Ts. These two components were normalized to a 0–1 scale and multiplied to generate the FTS (20).

Bacterial and fungal OTUs that appeared and disappeared across the four seasonal freeze-thaw periods and between the two depths were quantified to assess species turnover. First, we filtered out OTUs with an abundance of ≥50 reads (0.1%) and defined them as present OTUs. Subsequently, we recorded the presence or absence of each OTU relative to the preceding period. OTUs were classified as “appeared” (if absent previously) or “disappeared” (if present previously). To visualize microbial community variation across periods and depths, PCoA was conducted in R with the “ape” and “vegan” packages, using both unweighted and weighted UniFrac distance matrices (65, 66). Weighted UniFrac distance considers both species composition and relative abundances, whereas unweighted UniFrac distance relies solely on presence-absence information (67). Furthermore, beta diversity decomposition was employed to examine the relative contributions of species turnover and nestedness using the “beta.div.comp” function in the “adespatial” R package (68).

To evaluate the relative influence of various ecological processes governing microbial community assembly throughout the seasonal freeze-thaw periods and between the two soil depths, we employed the phylogenetic bin-based null model analysis via the “iCAMP” R package (69). This method assesses the contribution of five distinct assembly processes by partitioning microbial taxa into phylogenetic bins: homogeneous selection, heterogeneous selection, dispersal limitation, homogenizing dispersal, and drift (69). The results were graphically represented by the Interactive Tree of Life (70). In addition, we utilized the neutral community model (NCM) to evaluate the relationship between taxa abundance and its frequency of occurrence, implemented with the “Hmisc,” “minpack.lm,” and "stats4" R packages (71). This NCM estimated a migration rate parameter, which calculates the probability that a randomly lost individual in a local community was replaced by dispersal from the metacommunity.

Distance-decay relationships for microbial communities were analyzed by regressing community similarity (1, dissimilarity) against environmental distance calculated as the Euclidean distance of standardized environmental factors, employing the R package “vegan” (65). Correlations between microbial assembly processes and environmental factors were assessed using a modified Mantel test approach after first removing variables with strong collinearity (Fig. S9). To identify the best-fitting models, we performed logarithmic transformations on the assembly processes and environmental variables. Specifically, for assembly processes (*Y*) and each variable (*X*), we used either their raw values (*Y* ~ *X*), log-transformed one variable (*Y* ~ ln*X* or ln*Y* ~ *X*), or log-transformed both variables (ln*Y* ~ ln*X*) into the Mantel test model. Model selection was based on Mantel’s *r*. For logarithmic transformation, factors containing 0 or negative values were first adjusted by subtracting the minimum value from all observations. Any 0 values remaining after this adjustment were replaced with 5% of the smallest positive value (equivalent to −3.00 on the natural log scale) before transformation (69).

Co-occurrence relationships among phylogenetic bins were inferred using the sparse correlation (SparCC) approach, which is designed for compositional data. Only edges with an absolute SparCC correlation coefficient above a specified threshold (|*R*| > 0.6) and adjusted *P* < 0.05 were retained in the final network (59). The resulting network was visualized with Gephi, an interactive network analysis platform (v0.10.1) (72).

SEM was employed to assess the direct and indirect effects of environmental variables, community assembly processes, beta diversity, and network cluster on WSOCD. The soil properties were represented by the first principal component, with collinear variables removed (Fig. S9). Model goodness-of-fit was evaluated against multiple criteria: the χ^2^ test (acceptable range: 0 ≤ χ^2^/df ≤ 2 and 0.05 ≤ *P* ≤ 1.00), the root mean square error of approximation (RMSEA, 0.00 ≤ RMSEA ≤ 0.05), and the comparative fit index, with values approaching 1 indicating a strong fit. In our study, SEM was used as an exploratory, process-based path model to evaluate whether a hypothesized ecological pathway is consistent with the covariance structure of the data, rather than as a strict test of causal dominance among community-derived metrics. To assist with SEM interpretation, the standardized effects of each variable on WSOCD were calculated. Path coefficients were used to describe both the magnitude and direction of these relationships. The SEM was carried out using AMOS 26.0 (Amos Development Corporation).

Differences in beta diversity and WSOCD across the four freeze-thaw periods at each depth were determined using one-way analysis of variance. Additionally, differences between the topsoil and subsoil layers across all sampling periods were evaluated using Student’s *t*-tests.

## Data Availability

The sequencing data supporting this study are available under CNSA (https://db.cngb.org/cnsa/) of CNGBdb with accession code CNP0001093, which were originally published in Chen et al. (21). For the bacterial data set, both raw paired-end reads and merged sequences are provided. For the fungal data set, merged sequences are deposited.

## SUPPLEMENTAL MATERIAL

Supplemental material msystems.00067-26-s0001.docx Supplemental figures, tables, and text.

ASM does not own the copyrights to Supplemental Material that may be linked to, or accessed through, an article. The authors have granted ASM a non-exclusive, world-wide license to publish the Supplemental Material files. Please contact the corresponding author directly for reuse.
