# Supplementary material for: Freeze-thaw strength regulates carbon density through microbial assembly processes and network clustering
Source: mSystems. 2026 May 22;11(6):e00067-26. doi: 10.1128/msystems.00067-26 (PMC13289174; doi:10.1128/msystems.00067-26)
Supplement: Supplemental material — Supplemental figures, tables, and text. [file msystems.00067-26-s0001.docx]

**Supplementary material**

**Supplementary Figures**


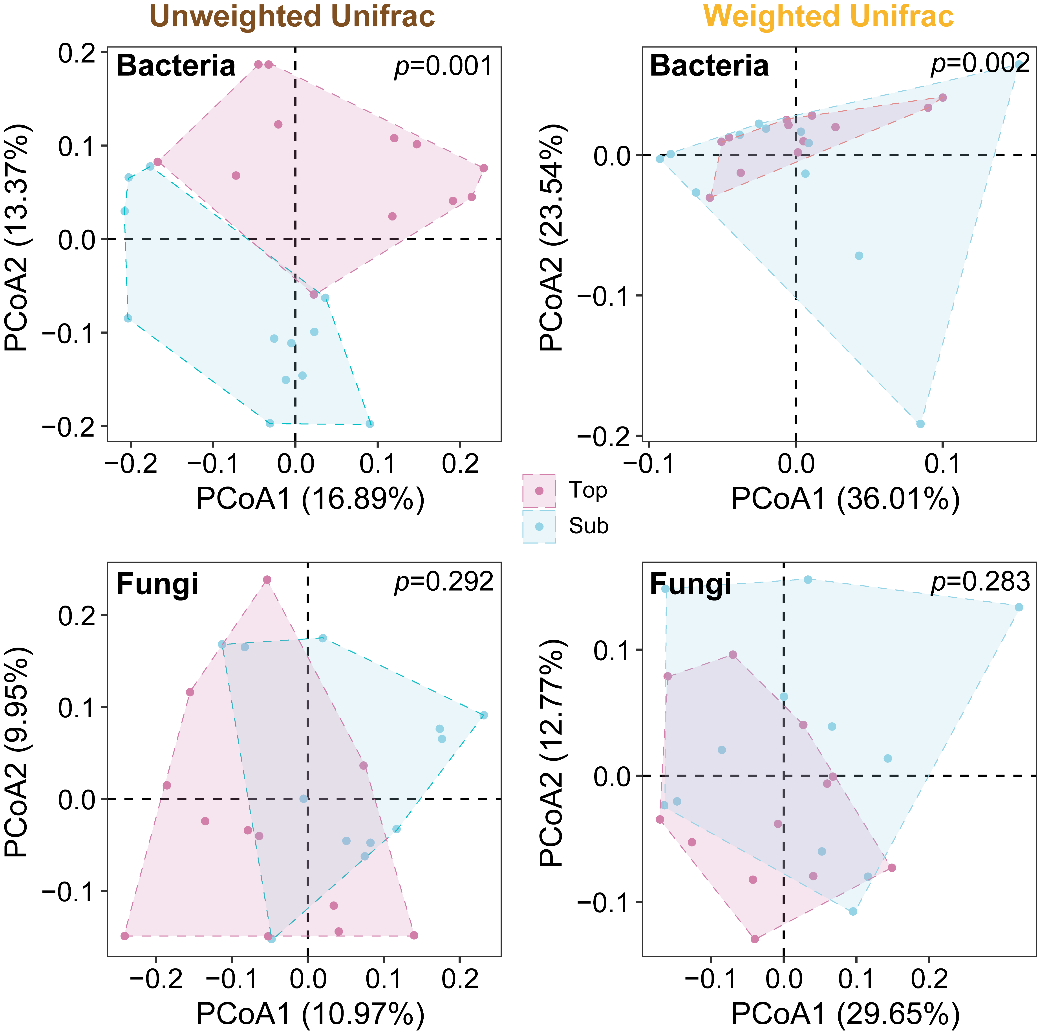


**Fig. S1.** **Vertical variations of composition in microbial communities at two depths.** PCoA and PERMANOVA on bacterial and fungal communities at two depths based on unweighted and weighted Unifrac distances.


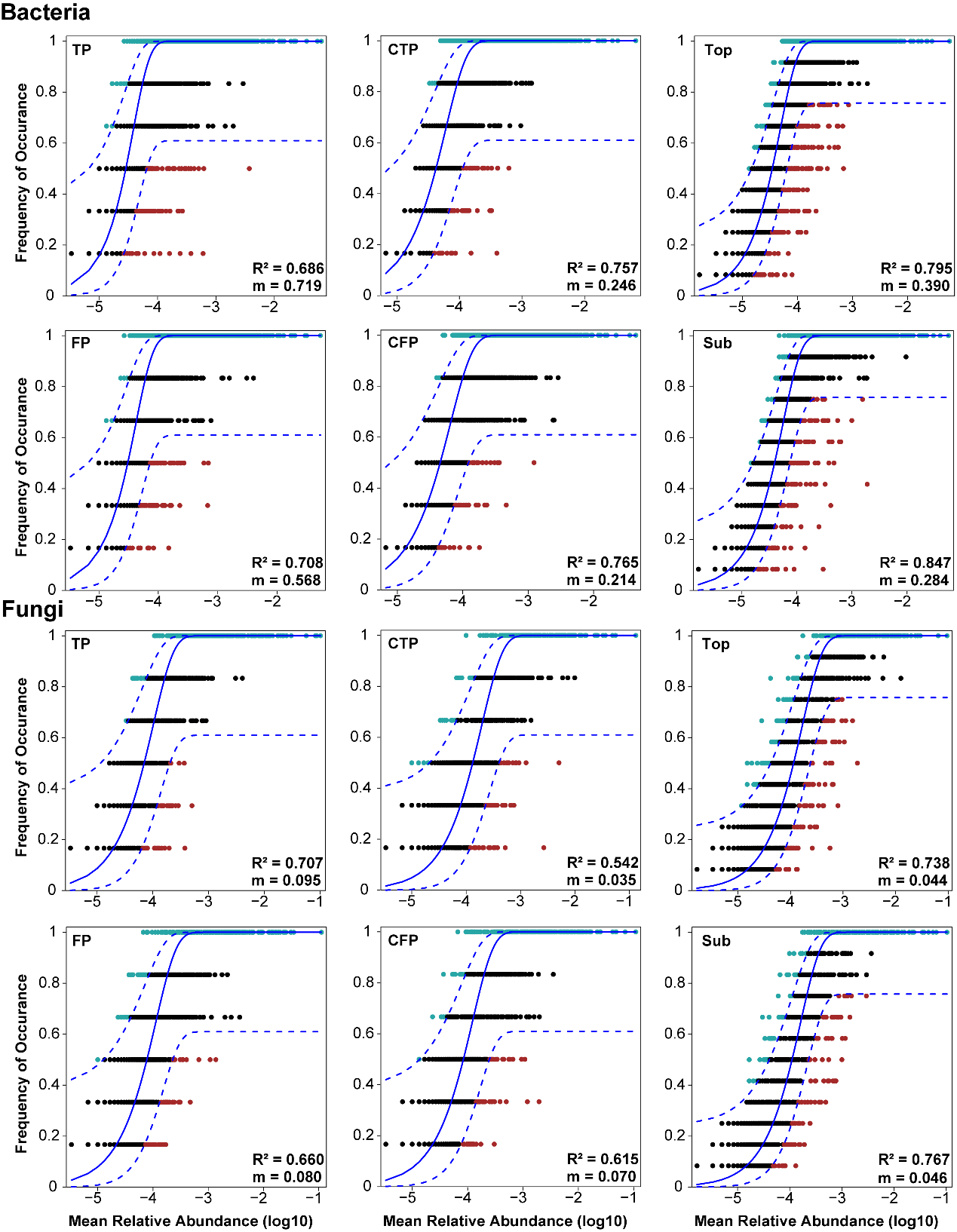


**Fig. S2. Neutral models of microbial communities during four periods of seasonal freeze-thaw processes and at two depths.** Neutral model analysis of assembly processes in bacterial and fungal communities in different periods and depths. The solid blue line represents the best-fitting neutral model. The dashed line represents the 95% confidence intervals around the best-fitting neutral model. OTUs within the black points follow the neutral process. OTUs that occur more frequently than predicted by the model are shown in green, whereas those that occur less frequently than predicted are shown in red. *m* indicates the estimated migration rate, and *R*^2^ indicates the fit to the neutral model.


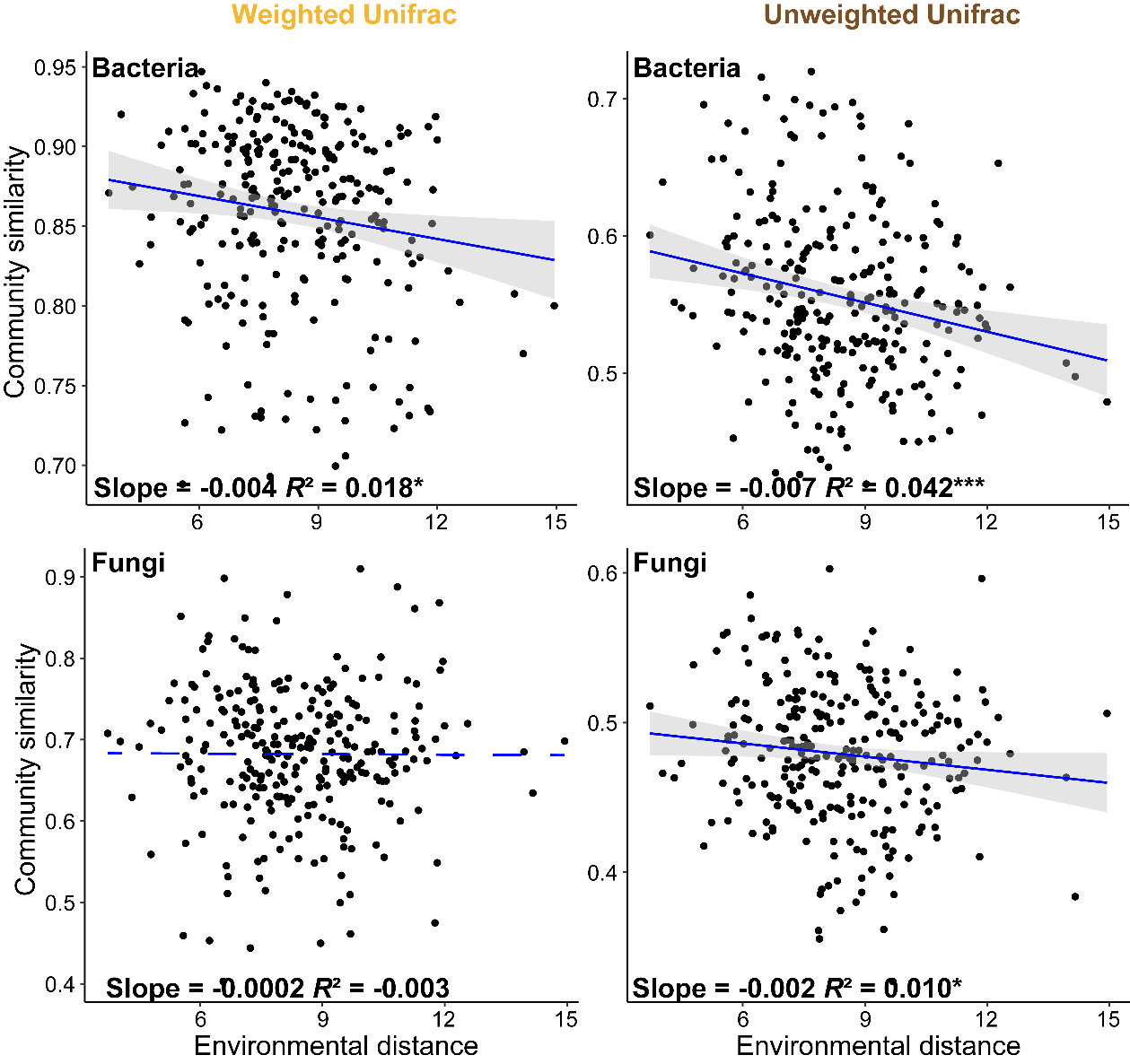


**Fig. S3.** **Distance-decay relationships between microbial community** **similarity and environmental distance.** Bacterial and fungal community similarity based on unweighted and weighted Unifrac distances is shown in relation to environmental distance. Asterisks indicate statistical significance (*** *p* < 0.001, ** *p* < 0.01, and * *p* < 0.05).

**
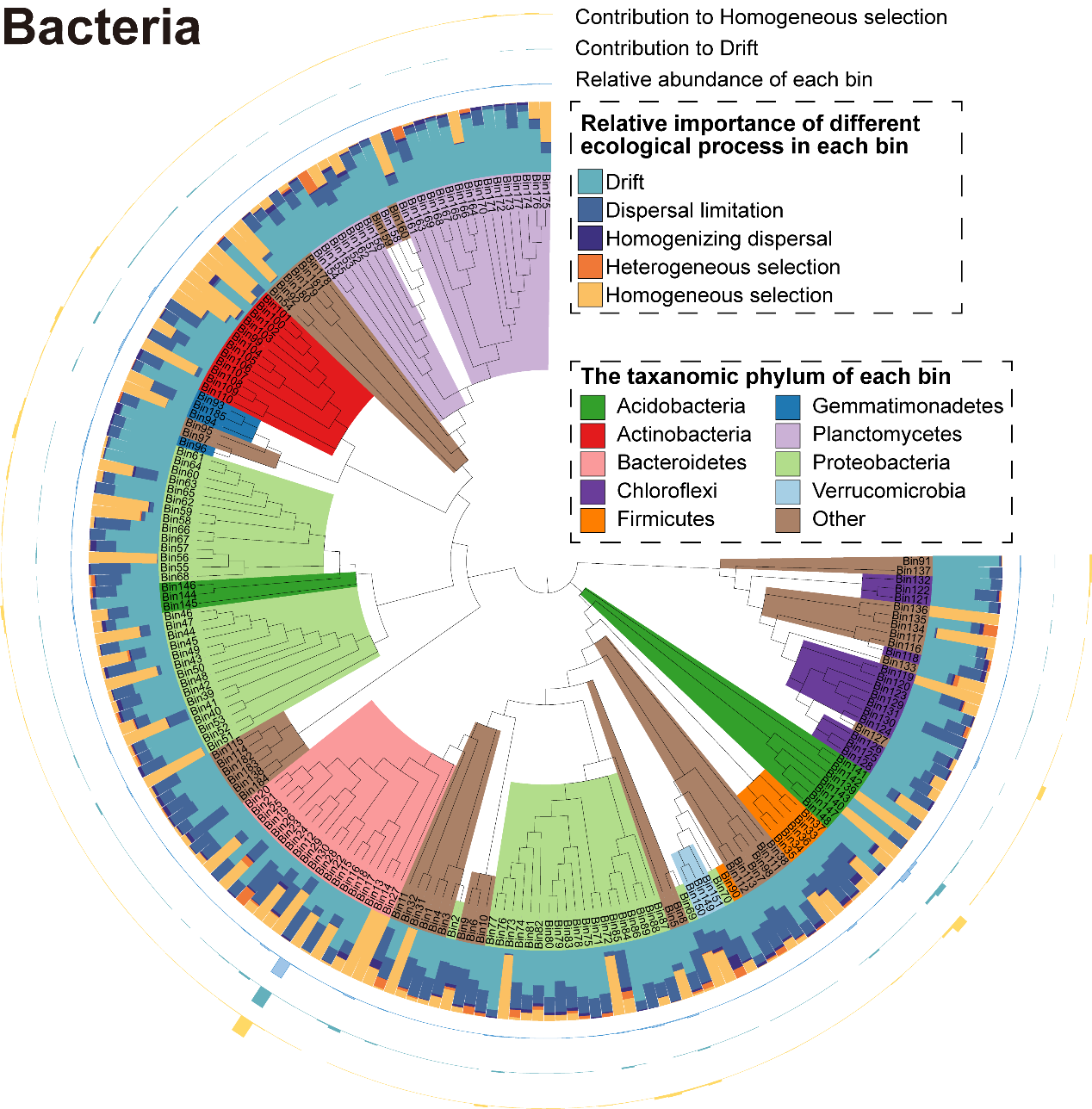
**

**Fig. S4. Ecological processes across phylogenetic groups (bins).** Relative importance of different assembly processes across bins in bacterial communities. Phylogenetic tree is centrally positioned. The stacked bars in the first annulus show the relative importance of ecological processes in each bin. The 2nd annulus exhibits the relative abundance of each bin. The 3rd and 4th annuli portray the contributions of all bins to homogeneous selection and dispersal limitation, respectively.

**
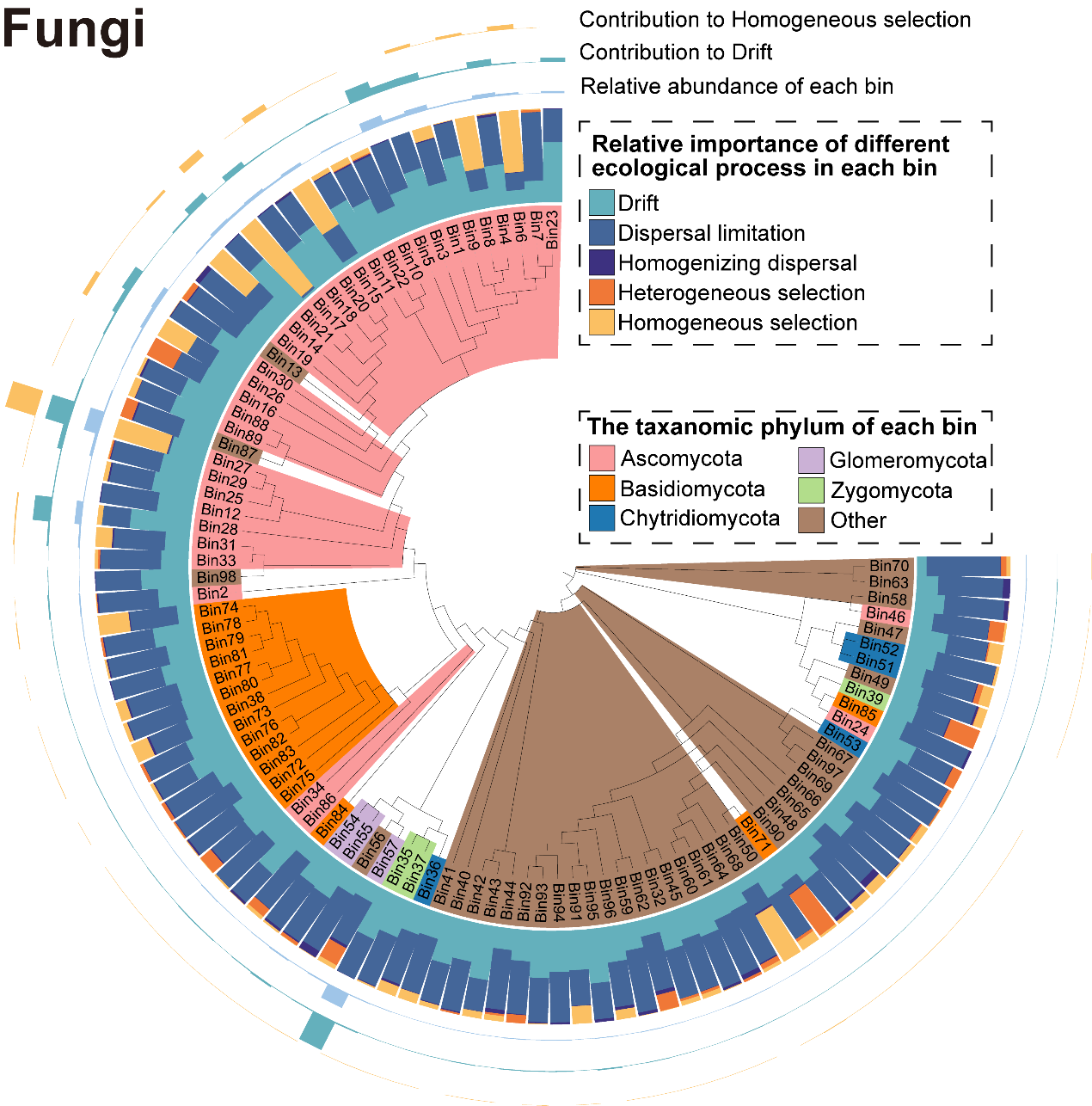
**

**Fig. S5. Ecological processes across phylogenetic groups (bins).** Relative importance of different assembly processes across bins in fungal communities. Phylogenetic tree is centrally positioned. The stacked bars in the first annulus show the relative importance of ecological processes in each bin. The 2nd annulus exhibits the relative abundance of each bin. The 3rd and 4th annuli portray the contributions of all bins to homogeneous selection and dispersal limitation, respectively.


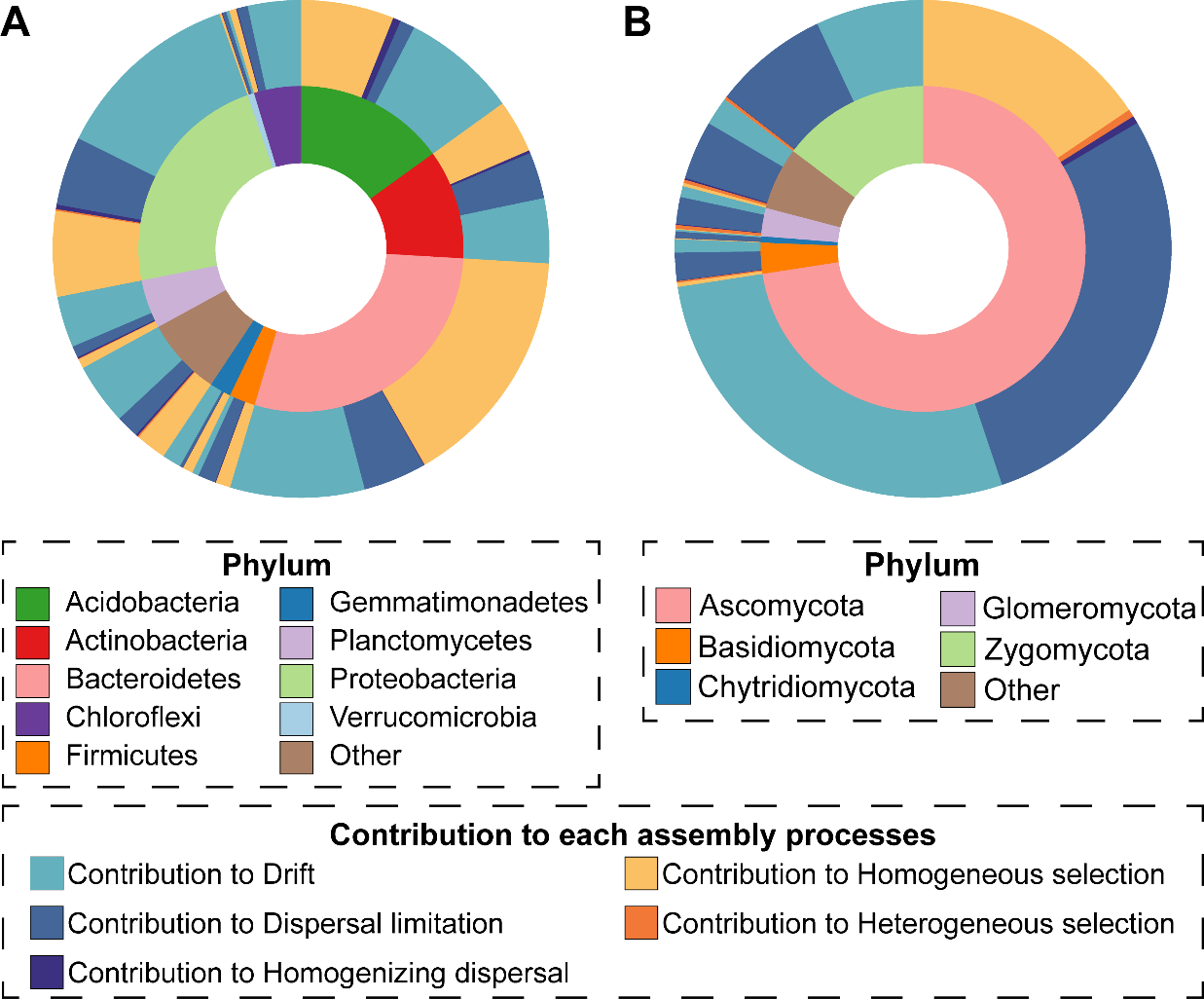


**Fig S6.** **The contributions of each phylum to assembly processes.** The 1st annulus shows the phylum in bacterial (**A**) and fungal (**B**) communities. The 2nd annulus exhibits the contributions of each phylum to each assembly processes.


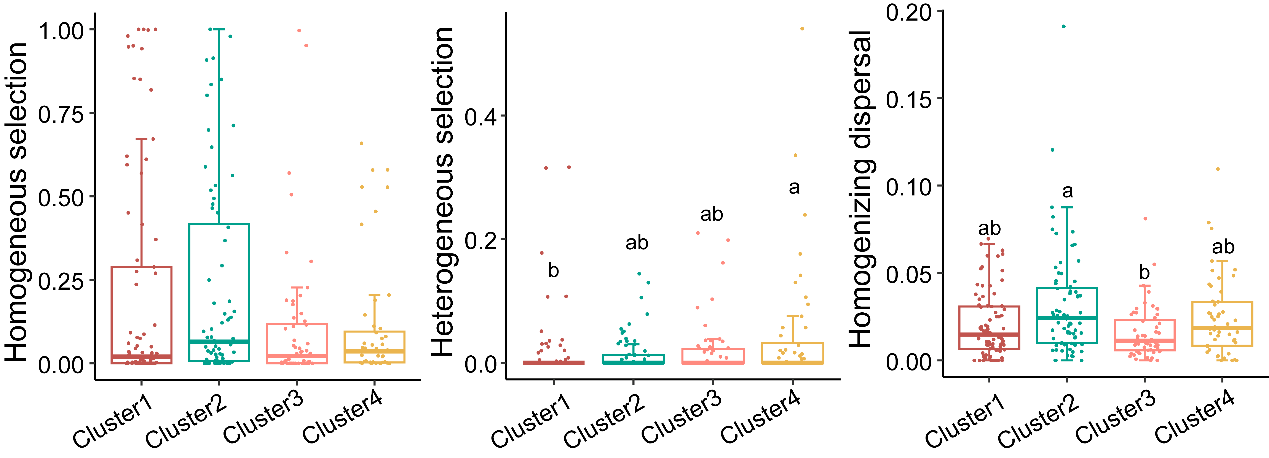


**Fig. S7.** **Differences in the assembly process of distinct microbial network clusters.** Lowercase letters represent the significance of differences among the four clusters (*p* < 0.05).


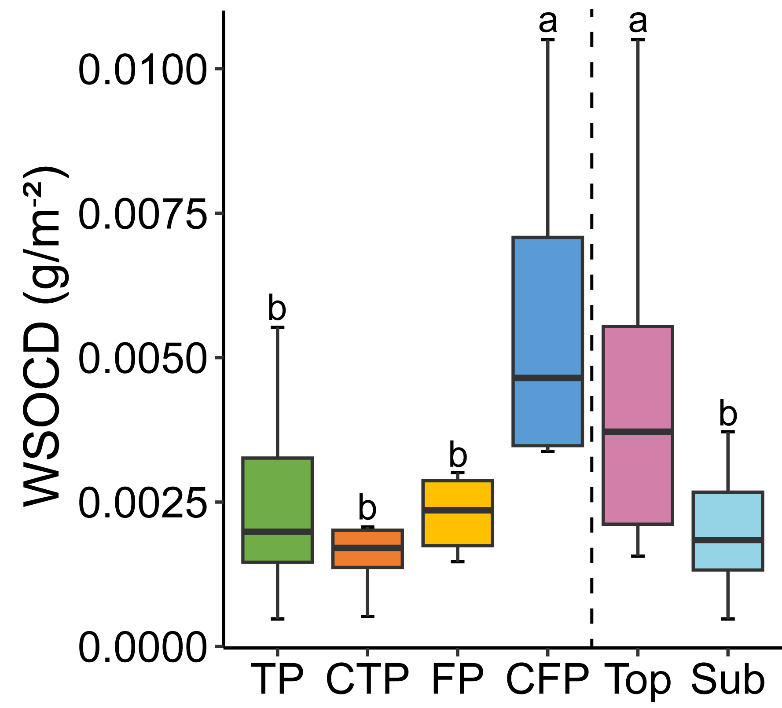


**Fig. S8.** **Variation patterns of WSOCD during four periods of seasonal freeze-thaw processes and at two depths.** Lowercase letters represent the significance of differences among the four periods or between the two depths (*p* < 0.05).


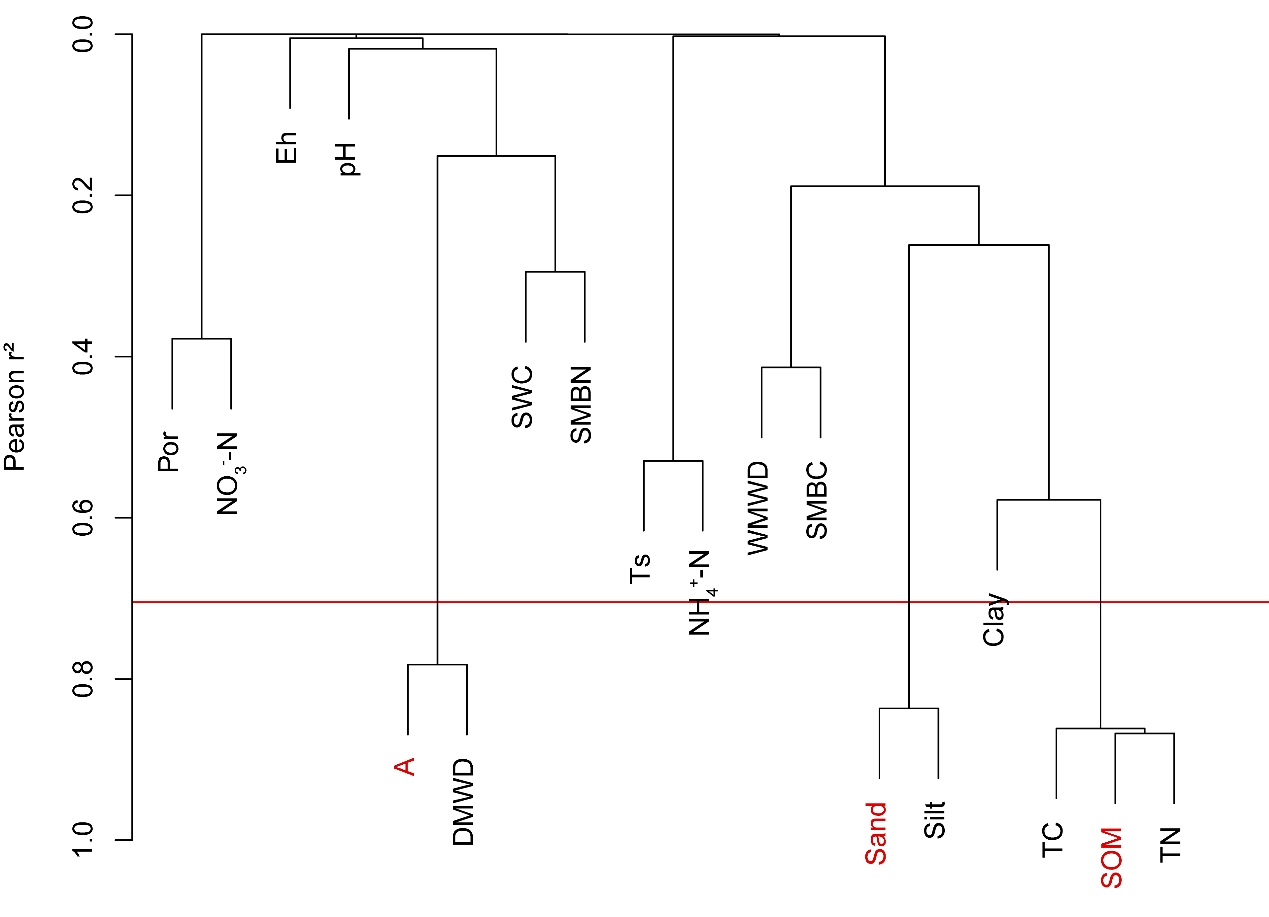


**Fig. S9.** **Cluster analysis of the measured environmental variables.** Variables with higher Pearson correlation (r^2^ > 0.7) are removed from the principal component analysis. The variables in red fonts are selectively retained. Ts, soil temperature; SWC, soil water content; Eh, redox potential; Por, soil porosity; A, aggregate content; DMWD, dry-sieving mean weight diameter; WMWD, wet-sieving mean weight diameter; SOM, soil organic matter; TC, soil total carbon; SMBC, soil microbial biomass carbon; SMBN, soil microbial biomass nitrogen; BGB, belowground biomass.

**Supplementary Tables**

**Table S1. Pairwise PERMANOVA results for bacterial and fungal community composition across freeze-thaw periods.**

| Unweighted UniFrac distance | | | | | | |
| --- | --- | --- | --- | --- | --- | --- |
|  | Bacteria | | | Fungi | | |
| **Pairs** | **F** | ***R*^2^** | ***p*** | **F** | ***R*^2^** | ***p*** |
| TP vs CTP | 1.130 | 0.101 | 0.317 | 1.595 | 0.138 | 0.007 |
| TP vs FP | 0.840 | 0.078 | 0.644 | 1.251 | 0.111 | 0.078 |
| TP vs CFP | 1.706 | 0.146 | 0.018 | 1.198 | 0.107 | 0.073 |
| CTP vs FP | 0.633 | 0.059 | 0.890 | 1.387 | 0.122 | 0.024 |
| CTP vs CFP | 1.333 | 0.118 | 0.139 | 1.755 | 0.149 | 0.003 |
| FP vs CFP | 1.012 | 0.092 | 0.408 | 1.298 | 0.115 | 0.074 |
| Weighted UniFrac distance | | | | | | |
|  | Bacteria | | | Fungi | | |
| **Pairs** | **F** | ***R*^2^** | ***p*** | **F** | ***R*^2^** | ***p*** |
| TP vs CTP | 1.495 | 0.064 | 0.132 | 1.413 | 0.124 | 0.146 |
| TP vs FP | 1.183 | 0.106 | 0.309 | 0.806 | 0.075 | 0.600 |
| TP vs CFP | 0.814 | 0.075 | 0.577 | 1.439 | 0.126 | 0.166 |
| CTP vs FP | 1.875 | 0.158 | 0.075 | 1.131 | 0.102 | 0.302 |
| CTP vs CFP | 1.487 | 0.129 | 0.178 | 2.908 | 0.225 | 0.014 |
| FP vs CFP | 1.800 | 0.153 | 0.109 | 1.406 | 0.123 | 0.164 |

**Table S2 The node information of the microbial co-occurrence network.**

| **Network cluster** | **Bins** | **Bacterial nodes** | **Bacterial proportion** | **Fungal nodes** | **Fungal proportion** |
| --- | --- | --- | --- | --- | --- |
| 1 | 81 | 75 | 92.5% | 6 | 7.5% |
| 2 | 72 | 65 | 90.2% | 7 | 9.8% |
| 3 | 56 | 11 | 19.6% | 45 | 80.4% |
| 4 | 48 | 19 | 39.5% | 29 | 60.5% |

**Table S3. Network cluster membership and phylum-level affiliation of bacterial and fungal phylogenetic bins.**

| **Bin_ID** | **Kingdom** | **Network cluster** | **Phylum** |
| --- | --- | --- | --- |
| Bin10_B | Bacteria | 1 | Microgenomates |
| Bin100_B | Bacteria | 1 | Actinobacteria |
| Bin107_B | Bacteria | 1 | Actinobacteria |
| Bin108_B | Bacteria | 1 | Actinobacteria |
| Bin109_B | Bacteria | 1 | Actinobacteria |
| Bin110_B | Bacteria | 1 | Actinobacteria |
| Bin111_B | Bacteria | 1 | Other |
| Bin115_B | Bacteria | 1 | Other |
| Bin116_B | Bacteria | 1 | Other |
| Bin117_B | Bacteria | 1 | Other |
| Bin129_B | Bacteria | 1 | Chloroflexi |
| Bin132_B | Bacteria | 1 | Chloroflexi |
| Bin134_B | Bacteria | 1 | Other |
| Bin136_B | Bacteria | 1 | Other |
| Bin137_B | Bacteria | 1 | Other |
| Bin139_B | Bacteria | 1 | Acidobacteria |
| Bin140_B | Bacteria | 1 | Acidobacteria |
| Bin142_B | Bacteria | 1 | Acidobacteria |
| Bin143_B | Bacteria | 1 | Acidobacteria |
| Bin144_B | Bacteria | 1 | Acidobacteria |
| Bin146_B | Bacteria | 1 | Acidobacteria |
| Bin147_B | Bacteria | 1 | Acidobacteria |
| Bin148_B | Bacteria | 1 | Acidobacteria |
| Bin152_B | Bacteria | 1 | Planctomycetes |
| Bin153_B | Bacteria | 1 | Planctomycetes |
| Bin154_B | Bacteria | 1 | Planctomycetes |
| Bin156_B | Bacteria | 1 | Planctomycetes |
| Bin160_B | Bacteria | 1 | Other |
| Bin164_B | Bacteria | 1 | Planctomycetes |
| Bin165_B | Bacteria | 1 | Planctomycetes |
| Bin167_B | Bacteria | 1 | Planctomycetes |
| Bin168_B | Bacteria | 1 | Planctomycetes |
| Bin169_B | Bacteria | 1 | Planctomycetes |
| Bin17_B | Bacteria | 1 | Bacteroidetes |
| Bin171_B | Bacteria | 1 | Planctomycetes |
| Bin173_B | Bacteria | 1 | Planctomycetes |
| Bin174_B | Bacteria | 1 | Planctomycetes |
| Bin175_B | Bacteria | 1 | Planctomycetes |
| Bin176_B | Bacteria | 1 | Planctomycetes |
| Bin177_B | Bacteria | 1 | Planctomycetes |
| Bin179_B | Bacteria | 1 | Other |
| Bin180_B | Bacteria | 1 | Latescibacteria |
| Bin181_B | Bacteria | 1 | Other |
| Bin182_B | Bacteria | 1 | Other |
| Bin183_B | Bacteria | 1 | Other |
| Bin184_B | Bacteria | 1 | Other |
| Bin19_B | Bacteria | 1 | Bacteroidetes |
| Bin20_B | Bacteria | 1 | Bacteroidetes |
| Bin22_B | Bacteria | 1 | Bacteroidetes |
| Bin25_B | Bacteria | 1 | Bacteroidetes |
| Bin27_B | Bacteria | 1 | Bacteroidetes |
| Bin31_B | Bacteria | 1 | Other |
| Bin50_B | Bacteria | 1 | Proteobacteria |
| Bin51_B | Bacteria | 1 | Proteobacteria |
| Bin54_B | Bacteria | 1 | Other |
| Bin55_B | Bacteria | 1 | Proteobacteria |
| Bin6_B | Bacteria | 1 | Other |
| Bin60_B | Bacteria | 1 | Proteobacteria |
| Bin61_B | Bacteria | 1 | Proteobacteria |
| Bin62_B | Bacteria | 1 | Proteobacteria |
| Bin64_B | Bacteria | 1 | Proteobacteria |
| Bin65_B | Bacteria | 1 | Proteobacteria |
| Bin74_B | Bacteria | 1 | Proteobacteria |
| Bin87_B | Bacteria | 1 | Proteobacteria |
| Bin88_B | Bacteria | 1 | Proteobacteria |
| Bin89_B | Bacteria | 1 | Proteobacteria |
| Bin9_B | Bacteria | 1 | Cyanobacteria |
| Bin90_B | Bacteria | 1 | Firmicutes |
| Bin91_B | Bacteria | 1 | Nitrospirae |
| Bin92_B | Bacteria | 1 | Other |
| Bin93_B | Bacteria | 1 | Gemmatimonadetes |
| Bin94_B | Bacteria | 1 | Gemmatimonadetes |
| Bin95_B | Bacteria | 1 | Entotheonellaeota |
| Bin96_B | Bacteria | 1 | Gemmatimonadetes |
| Bin99_B | Bacteria | 1 | Actinobacteria |
| Bin16_F | Fungi | 1 | Ascomycota |
| Bin26_F | Fungi | 1 | Ascomycota |
| Bin27_F | Fungi | 1 | Ascomycota |
| Bin35_F | Fungi | 1 | Zygomycota |
| Bin37_F | Fungi | 1 | Zygomycota |
| Bin45_F | Fungi | 1 | Other |
| Bin101_B | Bacteria | 2 | Actinobacteria |
| Bin102_B | Bacteria | 2 | Actinobacteria |
| Bin103_B | Bacteria | 2 | Actinobacteria |
| Bin104_B | Bacteria | 2 | Actinobacteria |
| Bin105_B | Bacteria | 2 | Actinobacteria |
| Bin106_B | Bacteria | 2 | Actinobacteria |
| Bin11_B | Bacteria | 2 | Other |
| Bin112_B | Bacteria | 2 | Cyanobacteria_Chloroplast |
| Bin113_B | Bacteria | 2 | Cyanobacteria_Chloroplast |
| Bin114_B | Bacteria | 2 | Other |
| Bin118_B | Bacteria | 2 | Chloroflexi |
| Bin125_B | Bacteria | 2 | Chloroflexi |
| Bin13_B | Bacteria | 2 | Bacteroidetes |
| Bin138_B | Bacteria | 2 | Armatimonadetes |
| Bin14_B | Bacteria | 2 | Bacteroidetes |
| Bin141_B | Bacteria | 2 | Acidobacteria |
| Bin149_B | Bacteria | 2 | Verrucomicrobia |
| Bin150_B | Bacteria | 2 | Verrucomicrobia |
| Bin151_B | Bacteria | 2 | Verrucomicrobia |
| Bin155_B | Bacteria | 2 | Planctomycetes |
| Bin158_B | Bacteria | 2 | Planctomycetes |
| Bin159_B | Bacteria | 2 | Other |
| Bin161_B | Bacteria | 2 | Planctomycetes |
| Bin163_B | Bacteria | 2 | Planctomycetes |
| Bin170_B | Bacteria | 2 | Planctomycetes |
| Bin172_B | Bacteria | 2 | Planctomycetes |
| Bin178_B | Bacteria | 2 | Other |
| Bin18_B | Bacteria | 2 | Bacteroidetes |
| Bin2_B | Bacteria | 2 | Proteobacteria |
| Bin21_B | Bacteria | 2 | Bacteroidetes |
| Bin28_B | Bacteria | 2 | Bacteroidetes |
| Bin29_B | Bacteria | 2 | Bacteroidetes |
| Bin3_B | Bacteria | 2 | Other |
| Bin30_B | Bacteria | 2 | Bacteroidetes |
| Bin34_B | Bacteria | 2 | Firmicutes |
| Bin35_B | Bacteria | 2 | Firmicutes |
| Bin38_B | Bacteria | 2 | Fibrobacteres |
| Bin40_B | Bacteria | 2 | Proteobacteria |
| Bin42_B | Bacteria | 2 | Proteobacteria |
| Bin44_B | Bacteria | 2 | Proteobacteria |
| Bin45_B | Bacteria | 2 | Proteobacteria |
| Bin46_B | Bacteria | 2 | Proteobacteria |
| Bin47_B | Bacteria | 2 | Proteobacteria |
| Bin48_B | Bacteria | 2 | Proteobacteria |
| Bin49_B | Bacteria | 2 | Proteobacteria |
| Bin5_B | Bacteria | 2 | Dependentiae |
| Bin56_B | Bacteria | 2 | Proteobacteria |
| Bin57_B | Bacteria | 2 | Proteobacteria |
| Bin58_B | Bacteria | 2 | Proteobacteria |
| Bin59_B | Bacteria | 2 | Proteobacteria |
| Bin63_B | Bacteria | 2 | Proteobacteria |
| Bin66_B | Bacteria | 2 | Proteobacteria |
| Bin67_B | Bacteria | 2 | Proteobacteria |
| Bin68_B | Bacteria | 2 | Proteobacteria |
| Bin70_B | Bacteria | 2 | Proteobacteria |
| Bin71_B | Bacteria | 2 | Proteobacteria |
| Bin72_B | Bacteria | 2 | Proteobacteria |
| Bin73_B | Bacteria | 2 | Proteobacteria |
| Bin75_B | Bacteria | 2 | Proteobacteria |
| Bin76_B | Bacteria | 2 | Proteobacteria |
| Bin78_B | Bacteria | 2 | Proteobacteria |
| Bin79_B | Bacteria | 2 | Proteobacteria |
| Bin83_B | Bacteria | 2 | Proteobacteria |
| Bin84_B | Bacteria | 2 | Proteobacteria |
| Bin86_B | Bacteria | 2 | Proteobacteria |
| Bin10_F | Fungi | 2 | Ascomycota |
| Bin13_F | Fungi | 2 | Ascomycota |
| Bin19_F | Fungi | 2 | Ascomycota |
| Bin20_F | Fungi | 2 | Ascomycota |
| Bin24_F | Fungi | 2 | Ascomycota |
| Bin41_F | Fungi | 2 | Other |
| Bin75_F | Fungi | 2 | Basidiomycota |
| Bin119_B | Bacteria | 3 | Chloroflexi |
| Bin12_B | Bacteria | 3 | Bacteroidetes |
| Bin120_B | Bacteria | 3 | Chloroflexi |
| Bin121_B | Bacteria | 3 | Chloroflexi |
| Bin122_B | Bacteria | 3 | Chloroflexi |
| Bin130_B | Bacteria | 3 | Chloroflexi |
| Bin135_B | Bacteria | 3 | Other |
| Bin145_B | Bacteria | 3 | Acidobacteria |
| Bin162_B | Bacteria | 3 | Planctomycetes |
| Bin69_B | Bacteria | 3 | Proteobacteria |
| Bin97_B | Bacteria | 3 | Other |
| Bin1_F | Fungi | 3 | Ascomycota |
| Bin12_F | Fungi | 3 | Ascomycota |
| Bin17_F | Fungi | 3 | Ascomycota |
| Bin18_F | Fungi | 3 | Ascomycota |
| Bin21_F | Fungi | 3 | Ascomycota |
| Bin22_F | Fungi | 3 | Ascomycota |
| Bin23_F | Fungi | 3 | Ascomycota |
| Bin28_F | Fungi | 3 | Ascomycota |
| Bin30_F | Fungi | 3 | Ascomycota |
| Bin31_F | Fungi | 3 | Ascomycota |
| Bin33_F | Fungi | 3 | Ascomycota |
| Bin34_F | Fungi | 3 | Ascomycota |
| Bin38_F | Fungi | 3 | Basidiomycota |
| Bin4_F | Fungi | 3 | Ascomycota |
| Bin40_F | Fungi | 3 | Other |
| Bin42_F | Fungi | 3 | Other |
| Bin44_F | Fungi | 3 | Other |
| Bin46_F | Fungi | 3 | Ascomycota |
| Bin47_F | Fungi | 3 | Other |
| Bin5_F | Fungi | 3 | Ascomycota |
| Bin51_F | Fungi | 3 | Chytridiomycota |
| Bin52_F | Fungi | 3 | Chytridiomycota |
| Bin53_F | Fungi | 3 | Chytridiomycota |
| Bin54_F | Fungi | 3 | Glomeromycota |
| Bin55_F | Fungi | 3 | Glomeromycota |
| Bin57_F | Fungi | 3 | Glomeromycota |
| Bin7_F | Fungi | 3 | Ascomycota |
| Bin73_F | Fungi | 3 | Basidiomycota |
| Bin74_F | Fungi | 3 | Basidiomycota |
| Bin76_F | Fungi | 3 | Basidiomycota |
| Bin77_F | Fungi | 3 | Basidiomycota |
| Bin78_F | Fungi | 3 | Basidiomycota |
| Bin79_F | Fungi | 3 | Basidiomycota |
| Bin8_F | Fungi | 3 | Ascomycota |
| Bin82_F | Fungi | 3 | Basidiomycota |
| Bin84_F | Fungi | 3 | Basidiomycota |
| Bin85_F | Fungi | 3 | Basidiomycota |
| Bin88_F | Fungi | 3 | Ascomycota |
| Bin91_F | Fungi | 3 | Other |
| Bin92_F | Fungi | 3 | Other |
| Bin93_F | Fungi | 3 | Other |
| Bin94_F | Fungi | 3 | Other |
| Bin95_F | Fungi | 3 | Other |
| Bin96_F | Fungi | 3 | Other |
| Bin98_F | Fungi | 3 | Other |
| Bin124_B | Bacteria | 4 | Chloroflexi |
| Bin126_B | Bacteria | 4 | Chloroflexi |
| Bin127_B | Bacteria | 4 | Other |
| Bin131_B | Bacteria | 4 | Chloroflexi |
| Bin15_B | Bacteria | 4 | Bacteroidetes |
| Bin16_B | Bacteria | 4 | Bacteroidetes |
| Bin24_B | Bacteria | 4 | Bacteroidetes |
| Bin26_B | Bacteria | 4 | Bacteroidetes |
| Bin32_B | Bacteria | 4 | Ignavibacteriae |
| Bin33_B | Bacteria | 4 | Firmicutes |
| Bin36_B | Bacteria | 4 | Firmicutes |
| Bin37_B | Bacteria | 4 | Firmicutes |
| Bin4_B | Bacteria | 4 | Other |
| Bin41_B | Bacteria | 4 | Proteobacteria |
| Bin52_B | Bacteria | 4 | Proteobacteria |
| Bin7_B | Bacteria | 4 | Other |
| Bin77_B | Bacteria | 4 | Proteobacteria |
| Bin8_B | Bacteria | 4 | Other |
| Bin85_B | Bacteria | 4 | Proteobacteria |
| Bin14_F | Fungi | 4 | Ascomycota |
| Bin15_F | Fungi | 4 | Ascomycota |
| Bin2_F | Fungi | 4 | Ascomycota |
| Bin29_F | Fungi | 4 | Ascomycota |
| Bin3_F | Fungi | 4 | Ascomycota |
| Bin36_F | Fungi | 4 | Chytridiomycota |
| Bin39_F | Fungi | 4 | Zygomycota |
| Bin48_F | Fungi | 4 | Other |
| Bin49_F | Fungi | 4 | Other |
| Bin58_F | Fungi | 4 | Ascomycota |
| Bin59_F | Fungi | 4 | Other |
| Bin60_F | Fungi | 4 | Other |
| Bin61_F | Fungi | 4 | Other |
| Bin62_F | Fungi | 4 | Other |
| Bin63_F | Fungi | 4 | Other |
| Bin64_F | Fungi | 4 | Other |
| Bin65_F | Fungi | 4 | Other |
| Bin66_F | Fungi | 4 | Other |
| Bin67_F | Fungi | 4 | Other |
| Bin68_F | Fungi | 4 | Other |
| Bin69_F | Fungi | 4 | Other |
| Bin70_F | Fungi | 4 | Other |
| Bin71_F | Fungi | 4 | Basidiomycota |
| Bin80_F | Fungi | 4 | Basidiomycota |
| Bin86_F | Fungi | 4 | Ascomycota |
| Bin87_F | Fungi | 4 | Other |
| Bin9_F | Fungi | 4 | Ascomycota |
| Bin90_F | Fungi | 4 | Other |
| Bin97_F | Fungi | 4 | Other |

**Supplementary Text**

**Bioinformatic analysis**

Raw sequence data from each sample were first processed with QIIME (Quantitative Insights Into Microbial Ecology, v1.9.1) to discard low-quality reads. Subsequently, paired-end reads were merged into high-confidence tag sequences in FLASH (Fast Length Adjustment of Short reads, v1.2.11) based on their overlapping regions, and any reads that could not be joined because of lacking overlap were removed. These tag sequences were then clustered into operational taxonomic units (OTUs) using USEARCH (Ultra-fast sequence analysis, v7.0.1090) at a 97% similarity cutoff to obtain representative sequences for each OTU. After filtering out chimera sequences, the OTU representatives were taxonomically assigned by comparing them against the Greengenes_2013_5_99 (v201305) and UNITE (v7.2) reference databases using the RDP Classifier (v2.2), with a confidence threshold of 0.6. Finally, all tag sequences were mapped back to the OTU representative sequences to generate OTU abundance tables and corresponding species-level annotation tables.
